# Identification and affinity enhancement of T-cell receptor targeting a KRAS^G12V^ cancer neoantigen

**DOI:** 10.1038/s42003-024-06209-2

**Published:** 2024-04-29

**Authors:** Mengyu Zhang, Wei Xu, Lingjie Luo, Fenghui Guan, Xiangyao Wang, Pei Zhu, Jianhua Zhang, Xuyu Zhou, Feng Wang, Sheng Ye

**Affiliations:** 1 Frontiers Science Center for Synthetic Biology (Ministry of Education), Tianjin Key Laboratory of Function and Application of Biological Macromolecular Structures, School of Life Sciences, Tianjin University, 92 Weijin Road, Nankai District, Tianjin, 300072 China; 2 CAS Key Laboratory of Pathogenic Microbiology and Immunology, Institute of Microbiology, Chinese Academy of Sciences (CAS), Beijing, 100101 China; 3 Department of Savaid Medical School, University of Chinese Academy of Sciences (CAS), Beijing, 100049 China; 4 School of Medicine, Shanghai University, Shanghai, 200444 China; 5 The Cancer Hospital of the University of Chinese Academy of Sciences, Institute of Basic Medicine and Cancer (IBMC), Chinese Academy of Sciences, Hangzhou, 310022 China; 6 State Key Laboratory of Oncogenes and Related Genes, Department of Immunology and Microbiology, Shanghai Jiao Tong University School of Medicine, Shanghai, 200025 China

**Keywords:** X-ray crystallography, Adaptive immunity

## Abstract

Neoantigens derived from somatic mutations in Kirsten Rat Sarcoma Viral Oncogene Homolog (KRAS), the most frequently mutated oncogene, represent promising targets for cancer immunotherapy. Recent research highlights the potential role of human leukocyte antigen (HLA) allele A*11:01 in presenting these altered KRAS variants to the immune system. In this study, we successfully generate and identify murine T-cell receptors (TCRs) that specifically recognize KRAS_8–16_^G12V^ from three predicted high affinity peptides. By determining the structure of the tumor-specific 4TCR2 bound to KRAS^G12V^-HLA-A*11:01, we conduct structure-based design to create and evaluate TCR variants with markedly enhanced affinity, up to 15.8-fold. This high-affinity TCR mutant, which involved only two amino acid substitutions, display minimal conformational alterations while maintaining a high degree of specificity for the KRAS^G12V^ peptide. Our research unveils the molecular mechanisms governing TCR recognition towards KRAS^G12V^ neoantigen and yields a range of affinity-enhanced TCR mutants with significant potential for immunotherapy strategies targeting tumors harboring the KRAS^G12V^ mutation.

## Abstract (web summary)

Neoantigens derived from somatic mutations in KRAS represent promising targets for cancer immunotherapy. Through structure-based design, the authors identify the KRAS-G12V specific affinity-enhanced TCR, offering great promise for advancing cancer immunotherapy.

## Introduction

KRAS is a member of the Ras family of GTPases, pivotal in transmitting signals from the extracellular milieu to intracellular phosphorylation cascades that control cellular growth, division, and differentiation^1^. KRAS functions as a molecular switch, oscillating between an inactive GDP-bound state and an active GTP-bound state. Its regulation primarily involves guanine exchange factors (GEFs), which promote the loading of GTP, and GTPase-activating proteins (GAPs), which facilitate the hydrolysis of GTP to GDP^2,3^. Single-point mutations in key residues of KRAS are capable of disrupting GTP hydrolysis by blocking interactions with GAPs and reducing the intrinsic GTPase activity of Ras, thereby trapping KRAS in the GTP-bound “on” state and leading to constitutive pro-growth signaling^4^.

KRAS stands out as the most frequently mutated oncogene, underlying the pathogenesis of ~20% of human cancer^5–8^. The most prevalent mutations occur at glycine residue 12 (G12) of KRAS, followed by glycine 13 (G13)^9^. Among the various KRAS mutations, KRAS^G12D^, KRAS^G12V^, and KRAS^G12C^, are the top three most frequently occurring alleles^5,10^. Notably, other KRAS alleles such as KRAS^G12S^ and KRAS^G13D^ are mainly restricted to colorectal cancer. However, the absence of an amenable mutant-specific binding pocket has hindered the development of selective small molecule inhibitor for KRAS^11^. Nevertheless, a breakthrough came with the discovery of covalent inhibitors targeting KRAS^G12C^, such as AMG510 (Sotorasib) and MRTX849 (Adagrasib), which have shown promising anti-tumor efficacy in clinical trials^12–14^. Furthermore, recent advancements introduced non-covalent inhibitors for KRAS^G12D^, namely MRTX1133, and TH-Z835, which are currently in preclinical stages^15–18^. However, no small molecule inhibitor targeting KRAS^G12V^, KRAS^G13D^, and KRAS^G12R^ has been reported thus far.

An alternative approach to target cancer-specific neoantigens, such as KRAS mutations, involves leveraging T-cell receptors (TCRs) for T-cell-mediated cancer immunotherapy^19^. Immune cells can recognize and eliminate cancer cells based on TCR recognition of neoantigen-derived peptides presented by human leucocyte antigen (HLA) class I on the cancer cell surface^20^. Because TCRs typically have moderate affinity for their peptide/Major Histocampatibility Complex (pMHC) ligands, there is a recognized need to develop affinity-enhanced TCR variants. These enhanced TCRs can enable the creation of bispecific molecules that guide native T cells towards tumor cells^21^.

In this study, we employed NetMHCpan-4.0 predictions and UV-mediated peptide exchange to predict and evaluate the binding capacity of KRAS-derived peptides to HLA-A*11:01. Through experiments with HLA-A*11:01 transgenic mice, we successfully isolated an array of murine TCRs specific for the KRAS^G12V^ nonapeptide (VVGAVGVGK), from three high-affinity KRAS-derived peptides. Among these TCRs, we determined the crystal structure of the 4TCR2-HLA-A*11:01-KRAS_8–16_^G12V^ complex. Using a structure-based design approach, we created variants of the 4TCR2 and subjected them to affinity assessments. We achieved a significant affinity enhancement with just two amino acid substitutions, located in CDR2β and CDR3β, respectively. Notably, the highest affinity variant exhibited no detectable interaction towards KRAS_8–16_^WT^ peptides presented by HLA-A*11:01. The structure of this high-affinity mutant TCR in complex with HLA-A*11:01-KRAS_8–16_^G12V^ revealed minimal conformational changes, highlighting the precision of our structure-guided design approach. The high affinity 4TCR2 variants identified in this study hold great promise for advancing cancer immunotherapy.

## Results

### Prediction and identification of KRAS-derived peptides

**Fig. 1:**
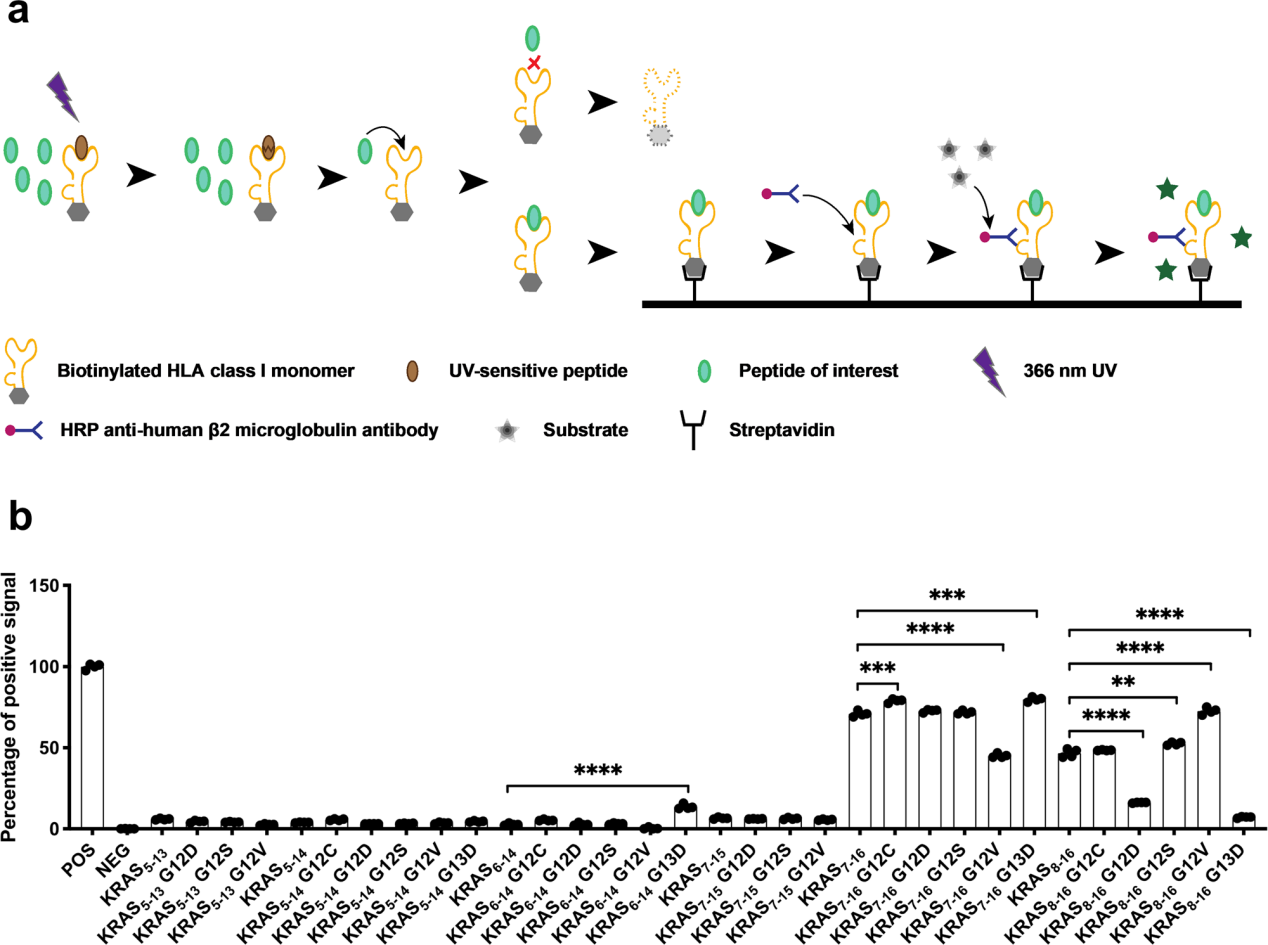
Assessment of predicted KRAS-derived neoantigen binding affinity to HLA-A*11:01 monomers. **a** UV-mediated HLA class I-peptide binding: UV-sensitive peptides, which are preloaded on biotinylated HLA class I monomers, are destroyed upon exposure to 366 nm UV light. This results in the exposure of the peptide-binding epitope on the biotinylated HLA class I monomers. Subsequently, co-incubated peptides of interest can readily bind to the exposed epitope. Biotinylated HLA class I monomers lacking bound peptide undergo degradation. The biotinylated HLA class I-peptide monomers bound to streptavidin anchors on the plate are then detected by using an HRP-conjugated anti-human β2 microglobulin antibody. The addition of a substrate leads to an enzymatic reaction with HRP, allowing for quantification of HLA class I-peptide monomers through OD measurement. **b** Interaction between preloaded UV-sensitive peptide-HLA-A*11:01 monomers and predicted KRAS-derived peptides: Upon exposure to UV light, the UV-sensitive peptides on HLA-A*11:01 monomers are degraded, thereby exposing the peptide-binding epitope on the HLA-A*11:01 monomers. Monomers lacking bound peptide are subsequently degraded. The evaluation of peptide-binding monomers is conducted using an Anti-human β2-microglobulin ELISA, with OD values serving as the basis for measurement. The capacity of peptide-monomer binding is expressed as the percentage of positive signal, calculated as follow: percentage of positive signal = [(OD value of monomers with the predicted peptide - OD value of monomers with the negative control peptide)/(OD value of monomers with the positive control peptide - OD value of monomers with the negative control peptide)] × 100%. Data are from one representative experiment out of three and are presented as mean ± SEM. For this experiment *n* = 4 biological replicates.*p < 0.05; **p < 0.005; ***p < 0.0005; ****p < 0.0001 as determined by the *t* test.

HLA-A*11:01 is the predominant Class I HLA allele in southern Chinese populations, with frequencies up to 40%^22,23^. It is also found at high frequencies in the United States, with approximately 14% in U.S. Caucasians and 23% in Asian-Americans^22^. We first employed the Promiscuous MHC Binding Peptide Prediction Server^24^ to analyze the wild-type and mutated KRAS protein sequences and identified 32 predicted KRAS-derived peptides exhibiting potential for HLA-A*11:01 binding capability (Supplementary Table 1). We next investigated the binding affinity of these predicted KRAS-derived peptides for HLA-A*11:01 by examining the stability of peptide-HLA-A*11:01 monomers in vitro. The approach involved incubating HLA class I monomers harboring UV-sensitive peptides with candidate peptides under UV exposure. As preloaded UV-sensitive peptides were degraded; candidate peptides with high affinity could efficiently load into the HLA-I binding groove, subsequently bolstering the stability of the peptide-HLA complex. Quantification of stable HLA-I complexes, now housing exchanged peptides, was carried out through ELISA employing an anti-β2 microglobulin antibody (Fig. 1a).

Using commercial UV-sensitive HLA-A*11:01 monomers, we proceeded to examine the HLA-A*11:01 binding capacity of the predicted KRAS-derived peptides. Our data underscore the following findings: KRAS_7-16_^WT^ (70.88%)/^G12C^ (78.95%)/^G12D^ (72.73%)/^G12S^ (71.78%)/^G12V^ (45.10%)/^G13D^ (79.83%) and KRAS_8–16_^WT^ (46.63%)/^G12C^ (48.50%)/^G12S^ (53.53%)/^G12V^ (72.60%) display a spectrum of binding affinities for HLA-A*11:01 monomers (Fig. 1b). Furthermore, KRAS_6-14_^G13D^ (13.60%) and KRAS_8–16_^G12D^ (16.18%) exhibited intermediate peptide-HLA-A*11:01 stability. In contrast, several other predicted KRAS-derived peptides exhibit significantly diminished HLA-A*11:01 binding capacity, with certain peptides rendering HLA-A*11:01 entirely undetectable. Our results indicated that the majority of KRAS-derived peptides boasting robust HLA-A*11:01 complex stability are located within KRAS_7-16_ and KRAS_8–16_ derived WT and mutations.

### Generation of mutated KRAS-reactive T cells with HLA-A*11:01 transgenic mice

**Fig. 2:**
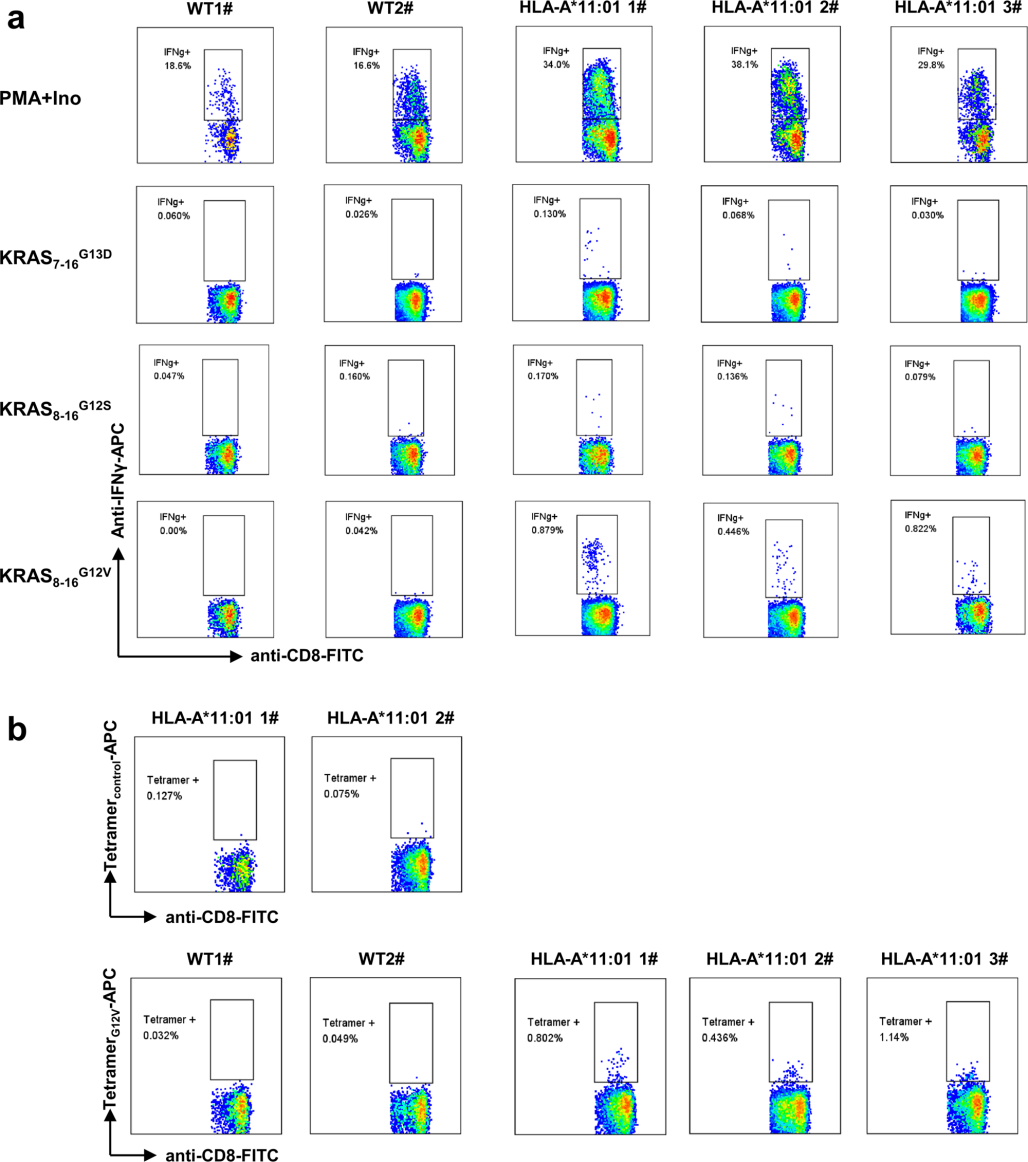
Immune response of HLA-A*11:01 transgenic mice to predicted neoantigens derived from high-affinity KRAS mutations. HLA-A*11:01 transgenic mice were subjected to an immunization regimen involving the top three KRAS-derived peptides with high HLA-A*11:01 affinity: KRAS_7–16_^G13D^, KRAS_8–16_^G12S^, and KRAS_8–16_^G12V^. This immunization occurred on both day 0 and day 14. Subsequently, on day 21, the mice received a reimmunization using plasmids carrying these three peptides. By day 28, peripheral blood samples were collected and subjected to separate stimulation with the aforementioned peptides. **a** The subsequent expression of IFNγ in CD8^+^ T cells was analyzed. **b** Based on the observations from Panel a, CD8^+^ T cells specific to KRAS_8–16_^G12V^ were identified and sorted using KRAS_8–16_^G12V^-HLA-A*11:01 tetramers. This experimentation utilized 2 wild-type and 3 HLA-A*11:01 transgenic mice, with PMA+Ino serving as the positive control.

To assess the immunogenicity of the screened peptides, we proceeded by utilizing the three KRAS-derived peptides with high affinity for HLA-A*11:01 molecules. These peptides were employed for immunization in HLA-A*11:01 transgenic mice as previously described^25^. Specifically, a mixture of KRAS_7-16_^G13D^, KRAS_8–16_^G12S^, and KRAS_8–16_^G12V^ peptides was subcutaneously administered twice to both WT and HLA-A*11:01 transgenic mice, once on day 0 and again on day 14. Subsequently, the mice received a re-immunization on day 21 with plasmids carrying the three peptides. On day 28, peripheral blood samples were collected and subjected to separate restimulation with the three peptides. Notably, in comparison to WT mice, HLA-A*11:01 transgenic mice exhibited significantly heightened responsiveness to all three peptides (Fig. 2a, Supplementary Fig. 1). Particularly, the HLA-A*11:01 transgenic mice displayed the highest Ifnγ expression response to KRAS_8–16_^G12V^, indicating that KRAS_8–16_^G12V^ could induce immune response in healthy mice and there are KRAS_8–16_^G12V^ specific CD8^+^ T cells. Thus, KRAS_8–16_^G12V^-HLA-A*11:01 tetramers were used to identify and sort the KRAS_8–16_^G12V^ peptide-specific CD8^+^ T cells of HLA-A*11:01 transgenic mice (Fig. 2b, Supplementary Fig. 2).

### Identification of HLA-A*11:01–restricted KRAS_8–16_^G12V^-reactive TCRs

**Fig. 3:**
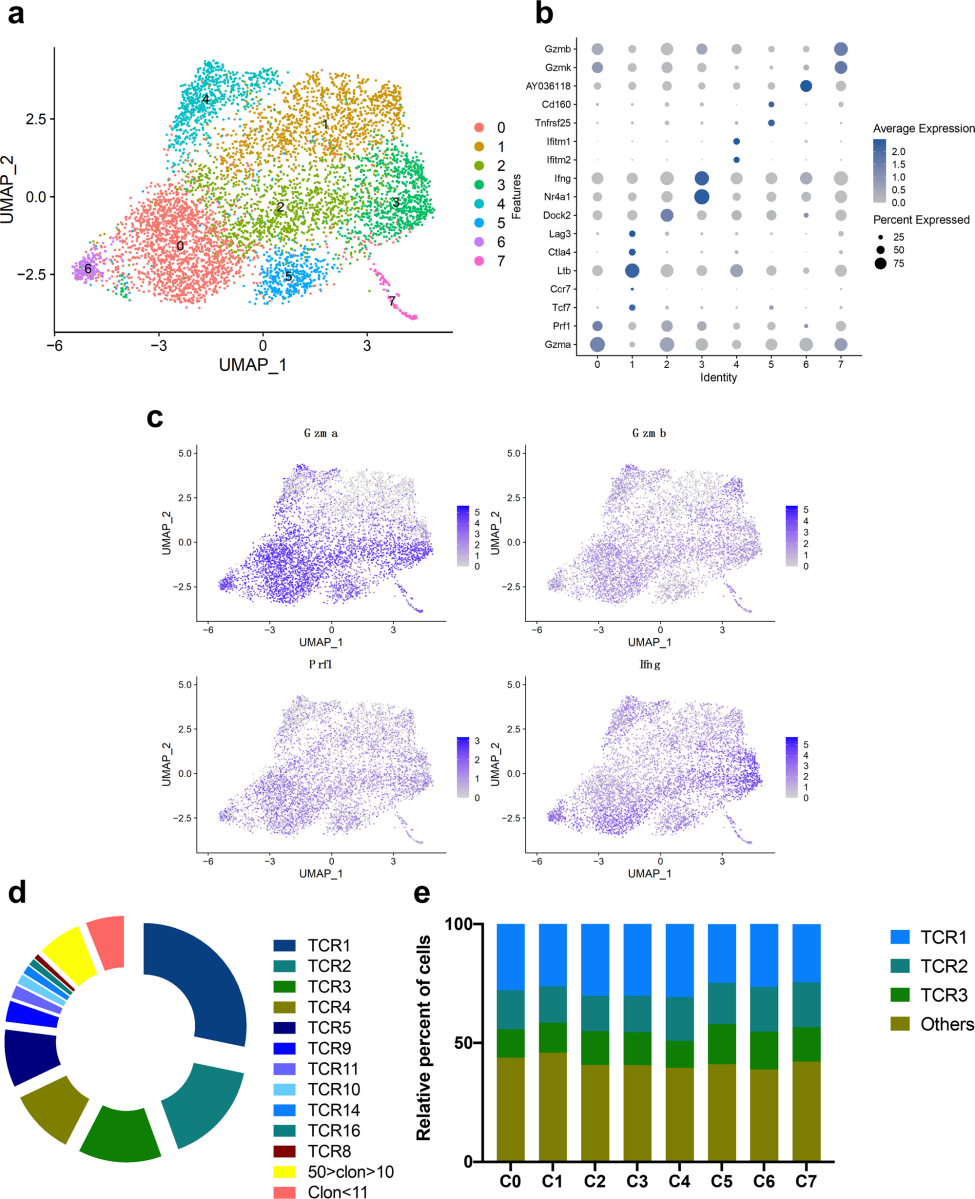
10× single-cell sequencing analysis. **a**, **b** Dimensionality reduction and clustering analysis of scRNA-seq data (**a**) and cluster annotation (**b**). **c** CD8^+^ T cells activation and effector marker expression analysis. **d** Distribution of highly expanded KRAS_8–16_^G12V^-specific CD8^+^ T cells clonotypes. **e** Distribution of the top 3 TCR clonotypes in each cluster. C0: Gzma^+^ Effector CD8 T cells, C1: Ltb^hi^ Exhausted CD8 T cells, C2: Dock2^+^ Effector CD8 T cells, C3: Ifng^hi^ Effector CD8 T cells, C4: Ifitm^hi^ Effector CD8 T cells, C5: Tnfrsf25^hi^ Effector CD8 T cells, C6: Proliferated CD8 T cells, C7: Gzmb^+^ Effector CD8 T cells.

To characterize these CD8^+^ T cells, we conducted 10× single-cell sequencing. We analyzed the RNA-seq data of CD8^+^ T cells specific to the KRAS_8–16_^G12V^ peptide using the Seurat Package. Our analysis of the single-cell transcriptomic data unveiled eight distinct subsets within these KRAS_8–16_^G12V^ peptide-specific CD8^+^ T cells: Gzma^+^ Effector CD8 T cells, Ltb^hi^ Exhausted CD8 T cells, Dock2^+^ Effector CD8 T cells, Ifng^hi^ Effector CD8 T cells, Ifitm^hi^ Effector CD8 T cells, Tnfrsf25^hi^ Effector CD8 T cells, Proliferated CD8 T cells and Gzmb^+^ Effector CD8 T cells (Fig. 3a, b). Notably, all of these clusters expressed markers of activated CD8^+^ T cells, including *Ifng*, as well as effector molecules Granzym (*Gzma*, *Gzmb*), and Perforin (*Prf1*) (depicted in Fig. 3c), which are capable of eliminating target cells. Concurrently, our TCR V(D)J analysis identified predominant TCR clonotypes within the KRAS_8–16_^G12V^ peptide-specific CD8^+^ T cells (Supplementary Table 2), expressed by a majority of these CD8^+^ T cells (Fig. 3d). Furthermore, the three most prominent TCR clonotypes accounted for over 50% of each cluster’s composition, as illustrated in Fig. 3e. These findings provide additional validation that the screened peptides, possessing high affinity with HLA-A*11:01, can effectively induced activation of CD8^+^ T cells, the pivotal effector immune cells in the fight against tumors.

### The crystal structure of TCR in complex with HLA-A*11:01-KRAS^G12V^

To investigate the molecular mechanisms underlying TCR selectivity for HLA-A*11:01-KRAS_8–16_^G12V^, our first step was to produce soluble and stable forms of these TCRs for further characterization and development. In essence, we introduced a non-native disulfide bond between the extracellular α and β constant domains, produced the TCRα and TCRβ chain inclusion bodies in *Escherichia coli,* and refolded them into soluble TCR forms. Out of the fourteen mTCRs attempted, two were successfully produced. These TCR variants exhibited remarkable stability and demonstrated genuine binding activity, as confirmed through BIAcore™ surface plasmon resonance (SPR) experiments.

**Fig. 4:**
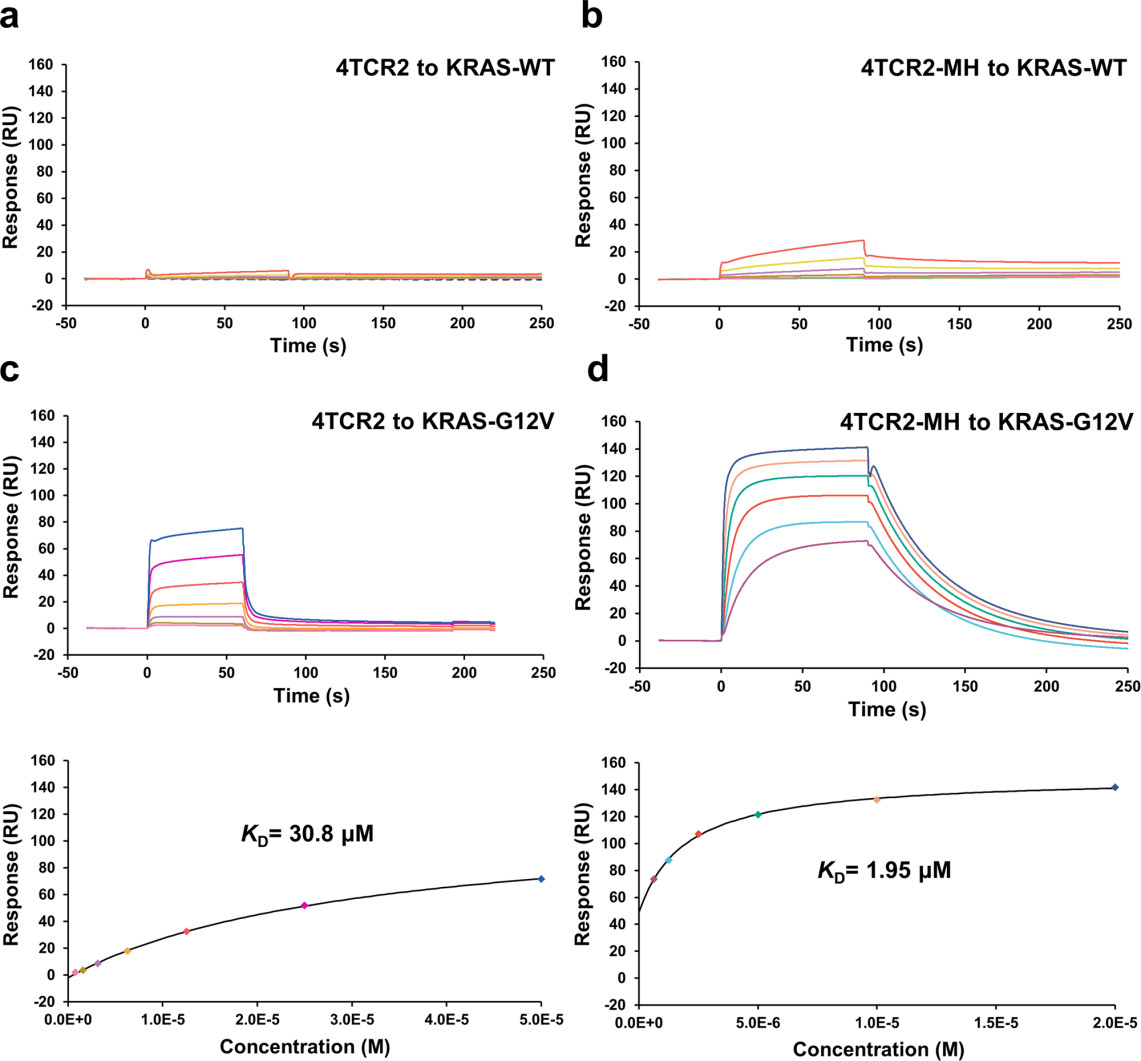
SPR analysis of TCR binding to KRAS^WT^–HLA-A*11:01 and KRAS^G12V^-HLA-A*11:01. **a** 4TCR2 at concentrations of 0.098, 0.195, 0.39, 0.78, 1.56, 3.12, 6.25, and 12.5 μM was injected over immobilized KRAS^WT^-HLA- A*11:01. **b** 4TCR2-MH at concentrations of 0.098, 0.195, 0.39, 0.78, 1.56, 3.12, 6.25, and 12.5 μM was injected over immobilized KRAS^WT^-HLA- A*11:01. **c** 4TCR2 at concentrations of 0.78, 1.56, 3.12, 6.25, 12.5, 25, and 50 μM was injected over immobilized KRAS^G12V^-HLA- A*11:01. **d** 4TCR2-MH at concentrations of 0.63, 1.25, 2.5, 5, 10, and 20 μM was injected over immobilized KRAS^G12V^-HLA- A*11:01.

To delve further, wild-type KRAS-HLA-A*11:01 or KRAS^G12V^-HLA-A*11:01 were selectively immobilized onto a CM5 biosensor surface. Over this immobilized pMHC ligand, varying concentrations of 4TCR2 flowed sequentially over the immobilized pMHC ligand. 4TCR2 were consecutively passed. The results displayed that 4TCR2 exhibited an affinity for KRAS^G12V^-HLA-A*11:01, with a dissociation constant (*K*_*D*_) of 30.8 μM (Fig. 4c). By contrast, no apparent interaction could be detected between 4TCR2 and KRAS^WT^-HLA-A*11:01 (Fig. 4a). The HLA-A*11:01-restricted 4TCR2 displayed its recognition of the KRAS^G12V^ neoepitope through the utilization of TRAV7D-2*01 and TRAJ22 for the α chain, and TRBV2 and TRBJ2-3 for the β chain (Supplementary Table 3). Notable, these gene segments are in contrast to previously identified TCRs targeting KRAS^G12V^^26,27^.

**Fig. 5:**
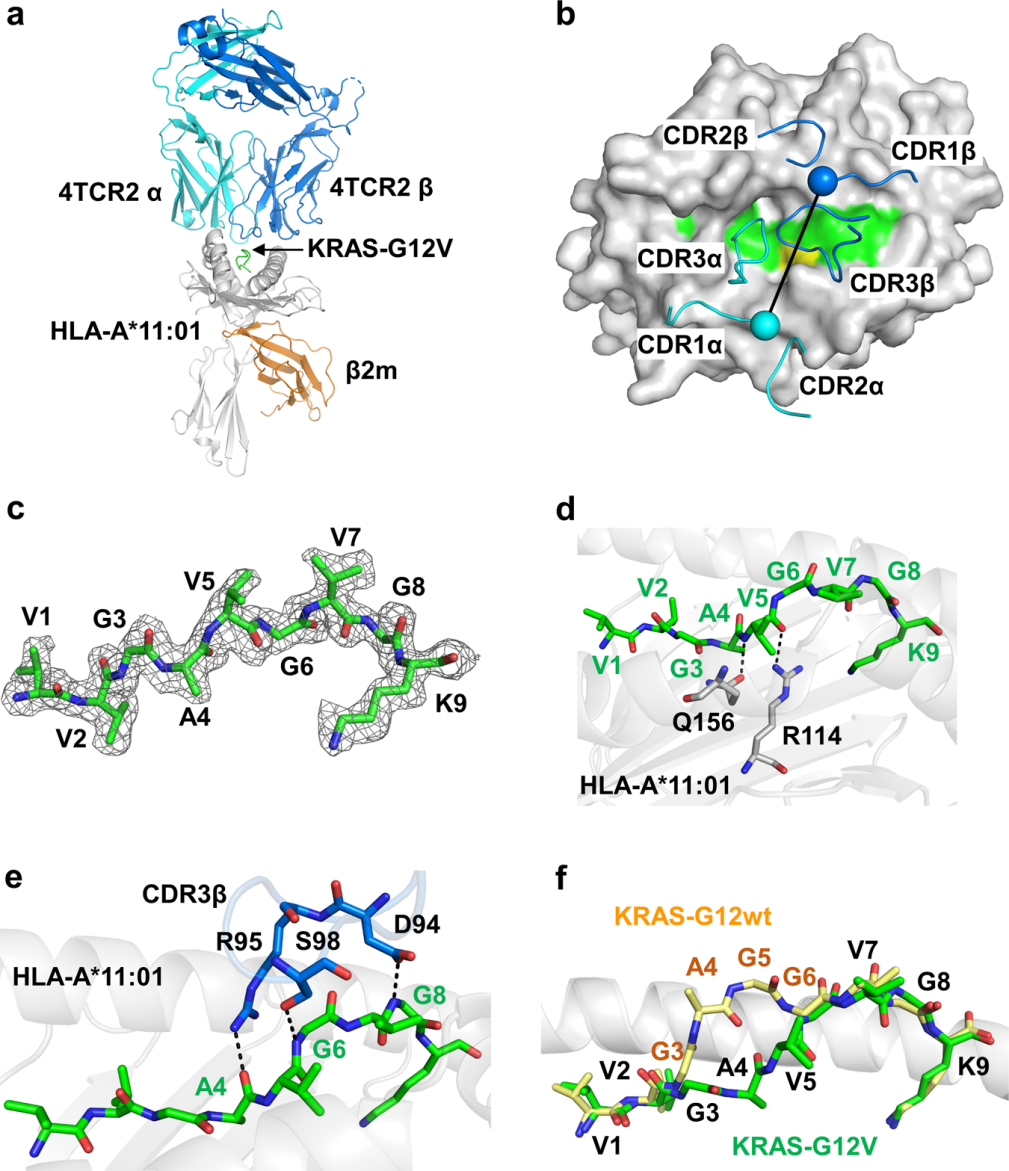
4TCR2 binds to the HLA-A*11:01 and the KRAS^G12V^ peptide. **a** Overall structure of KRAS^G12V^/HLA-A*11:01 bound to the 4TCR2 (PDB 8WTE). HLA-A*11:01 and β2 macroglobulin (β2m) are colored in gray and orange, respectively. TCR α chain and β chain are colored in cyan and blue, respectively. The KRAS^G12V^ nonapeptide is shown in green between helices α1 and α2 of the HLA. **b** Top view of the 4TCR2-HLA-A*11:01-KRAS^G12V^ complex. The HLA and KRAS^G12V^ peptide are shown in surface representation, and the CDRs are shown in cartoon tube representations. The crossing angle vector is drawn connecting the disulphides between the 4TCR2 TCR α chain (cyan sphere) and TCR β chain (blue sphere) variable domains. The KRAS-G12V peptide is presented as a surface in green with the mutated P5 Val residue in yellow. **c** Composite omit electron density map of the KRAS^G12V^ peptide contour at 1σ. **d** The HLA interaction network around the peptide residue V5 in the 4TCR2-HLA-A*11:01-KRAS^G12V^. **e** 4TCR2 CDR3β interactions with the KRAS^G12V^ peptide. **f** Comparison of the structure of KRAS^WT^ peptide (yellow) (PDB ID: 8I5E) with KRAS^G12V^ peptide (green) (PBD 8WTE) presented by HLA-A*11:01. The HLA helices are shown in cartoon.

To understand the structural basis of TCR specificity for HLA-A*11:01-KRAS_8–16_^G12V^, we purified the two complexes for crystallization trial. Subsequently, we determined the crystal structure of 4TCR2-HLA-A*11:01-KRAS_8–16_^G12V^ complex at a resolution of 2.17 Å (Fig. 5a, Supplementary Table 4). The electron density map exhibits well-resolved features for most amino acids, especially around the peptide-binding pocket, and clear density for KRAS_8–16_^G12V^ nonapeptide (Fig. 5c). There were two 4TCR2 and two HLA-A*11:01-KRAS_8–16_^G12V^ per asymmetric unit, with a pairwise root-mean-square deviation (RMSD) of 0.9 Å for 756 C^α^ carbons (Fig. 5a). Both 4TCR2s were firmed positioned on HLA-A*11:01-KRAS_8–16_^G12V^ with a total buried surface area of the interface calculated as 1676 Å^2^. When viewed along the axis from the N terminus to the C terminus of the KRAS_8–16_^G12V^ peptide, the docking angle of the 4TCR2 to the KRAS_8–16_^G12V^-pHLA was 62° (Fig. 5b). This orientation angle was in accordance with most previously described TCR-pMHC complexes^28^.

### Binding of the KRAS_8–16_^G12V^ peptide to HLA-A*11:01

The KRAS_8–16_^G12V^ peptide (VVGAVGVGK) occupied the binding groove α1-α2 of HLA-A*11:01, burying a solvent-accessible surface area of 2700 Å^2^, and with the C-terminal Lysine at position 9 (Lys 16) pointing down, inside of the groove. Moreover, the N terminus of the peptide was deeply situated within the peptide-binding groove, anchored by several residues in the HLA-A*11:01 (Supplementary Fig. 3a). The nonapeptide KRAS_8–16_^G12V^ adopted a canonical conformation within the cleft of HLA-A*11:01. The peptide contained anchor residues at P2-Val and P9-Lys. P2-Val forms a hydrogen bond with Glu63, while the side chain of P9-Lys is buried in the F pocket of HLA-A*11:01, forming a salt bridge with Asp116 and interacting with the hydrogen bonding networks formed by Tyr84, Tyr143, and Lys146 (Supplementary Fig. 3b, c). The side chains of the mutant Val 5 in the KRAS^G12V^ peptide extend toward the α2 helix of HLA-A*11:01, forming a hydrogen bond with Arg114 and Gln156 (Fig. 5d).

### Structural basis for the recognition of HLA-A*11-KRAS^G12V^ by the 4TCR2

The binding interface between HLA-A*11:01 and 4TCR2 involved all six complementarity-determining regions (CDRs), except CDR1β. The interactions between 4TCR2 and HLA*11:01 were primarily mediated by contacts between the TCR α-chain and the HLA α2 helix. Key residues of 4TCR2 at the HLA interface included Y49β, L52β, and M53β of CDR2β, that interacted with T73, R65, and Q62 of HLA α1, and R95β and S98β of CDR3β, that engaged with Q70 of the HLAα1 and Q155 of the HLA α2, respectively (Supplementary Fig. 4a). Additional interactions were observed through R28α of CDR1α, which formed salt bridges with E166 of HLA α2, and Y32α of CDR1α, S52α of CDR2α, R92α, and W97α of CDR3α, which contacted E154 and Q155 of HLA α2, as well as N66 of HLA α1 (Supplementary Fig. 4b). In contrast, only CDR3β interacted with the KRAS^G12V^ peptide. D94β, R95β, and S98β of CDR3β formed hydrogen bonds with G8, A4, and G6 of the KRAS^G12V^ peptide, respectively (Fig. 5e).

### An induced-fit binding mechanism for the 4TCR2 to KRAS^G12V^ peptides

To gain a deeper understanding of how 4TCR2 interacts with HLA-A*11:01-KRAS^G12V^, we conducted a comparative analysis between two complex structures: HLA-A*11:01-KRAS^WT^ (PBD: 8I5E)^26^ and TCR-bound 4TCR2-HLA-A*11:01-KRAS^G12V^ (PBD: 8WTE). Interestingly, we observed a significant difference in the conformation of the KRAS^WT^ peptides within the pMHC structures compared to its counterpart in the TCR-bound configurations. In the pMHC structures, the KRAS^WT^ peptide exhibited a unique conformation, where residues 4-6 formed a central bulge (Fig. 5f). Whereas, in the 4TCR2-bound form, we observed a substantial conformational shift, notably with peptide residue 6 in KRAS^G12V^ transitioning from an upward-facing exposed position to a downward-facing orientation (Fig. 5f). This pronounced alteration in conformation led to the interaction between the KRAS^G12V^ peptide and the HLA groove, forming a complex network of interactions with the HLA D-pocket (Fig. 5d). These observations strongly suggest that 4TCR2 binding induces a conformational change in the peptide, allowing the TCR to effectively engage with the HLA-A*11:01-KRAS^G12V^ complex. Furthermore, it became evident that the KRAS^G12V^ peptide could potentially establish additional bonds with the HLA groove, thereby enhancing the stability of the epitope when bound to the TCR.

### Design and affinities of TCR point mutants

One crucial requirement for the effectiveness and safety of T-cell immunotherapy in patients is the identification of an optimal range of TCR affinities. This range should promote efficient regression of tumors while minimizing the risk of autoimmune responses. Typically, due to the process of thymic selection, the physiological affinity of the TCR for pMHC molecules falls within a narrow range of 1 µM to 100 µM^29–31^. Various studies have established the affinity threshold for maximal T-cell activity, including anti-tumor T-cell responses, at 5–10 µM of peptide epitope^32–34^. In the case of the interaction between 4TCR2 and pMHC, the affinity is naturally weak (*K*_D_ = 30.8 μM). The inherent low affinity of TCRs imposes limitations on their therapeutic potential. Therefore, we employed structure-based computational design to enhance the affinity of the TCRs, thus rendering them valuable agents for targeting cancer.

We utilized the PyRosetta software tools^35^ to predict the affinity changes of 4TCR2 mutants for KRAS^G12V^/HLA-A*11:01. We generated point mutations for each residue within the complementarity-determining regions (CDRs) of the TCR. In total, we analyzed 988 substitutions of 52 residues from the 4TCR2 CDRs, which were subsequently ranked based on their predicted TCR-pMHC affinity. From these, we selected sixteen mutations for experimental testing. Mutagenesis was carried out using soluble gene constructs of 4TCR2, followed by expression and purification of the mutant proteins. The binding affinities of the mutants towards KRAS^G12V^/HLA-A*11:01 were then measured using surface plasmon resonance (Table 1).

**Table 1:** 4TCR2 mutants organized by design strategy and measured affinities toward KRAS^G12V^

Mutant	*K*_D_ (μM)	Fold change
wild-type	30.8	
αN29M	43.1	0.71
αN29T	33.1	0.93
αN29L	43.4	0.71
αS49R	38.5	0.80
αS49K	37.1	0.83
αS49Q	41.2	0.75
αS52Q	64.8	0.48
αS52I	56.7	0.54
αS52E	57.4	0.54
αS94A	60	0.51
αG95F	46.8	0.66
βK51M	11.3	2.73
βK51W	21.5	1.43
βE100V	8.14	3.78
βE100H	6.68	4.61
βE100M	24.2	1.27
βK51M-E100V	2.65	11.6
βK51M-E100H	1.95	15.8
βK51M-E100M	7.89	3.9

Source data are provided as Supplementary Data.

Out of the sixteen mutations tested, three demonstrated significantly improved affinities: βK51M, βE100V, and βE100H. Among all the mutants examined, the two βE100 mutants displayed the highest measured binding affinities (up to a 4.6-fold improvement for βE100H), while the βK51M mutant exhibited a 2.7-fold increase in affinity. Combining the affinity-enhancing mutations βK51M and βE100H (referred to as the MH double mutant) resulted in a substantial 15-fold improvement (from 30.8 μM to 1.95 μM) (Fig. 4d).

### Crystal structure of mutant 4TCR2 MH in complex with HLA-A*11:01-KRAS_8–16_^G12V^

**Fig. 6:**
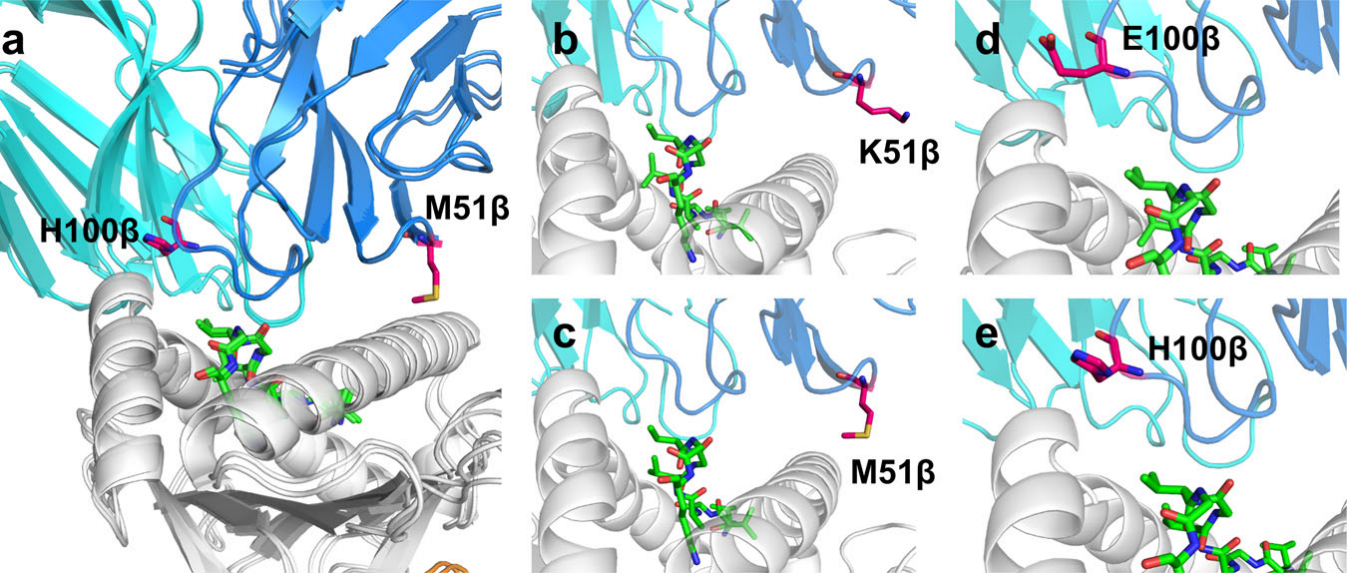
Structure of the 4TCR2 MH double mutant in complex with HLA-A*11:01-KRAS_8–16_^G12V^. **a** Superposition of the 4TCR2 MH/HLA-A*11:01-KRAS_8–16_^G12V^ and the 4TCR2/HLA-A*11:01-KRAS_8–16_^G12V^ complexes. 4TCR2 α chain is cyan, β chain is blue, MHC is gray, β2m is orange, and peptide is green (shown as sticks); residues that were mutated are shown as sticks. Close-ups of **b** wild-type K51β, **c** mutant M51β, **d** wild-type E100β, **e** mutant H100β are shown. **b**–**e** Residue of the mutation site is shown as stick.

To investigate the structural basis underlying the 15-fold improvement in binding affinity and compare it with the models generated during the design process, we conducted crystallization and structure determination of the 4TCR2 MH mutant in complex with HLA-A*11:01-KRAS_8–16_^G12V^, achieving a resolution of 2.36 Å (Fig. 6). Clear electron density was observed for the TCR-pMHC interface, and the positions of the mutated amino acids were unambiguous as indicated by an unbiased, iterative-build OMIT map (Supplementary Fig. 5). Similar to other engineered TCRs with high pMHC affinity that have been structurally characterized^36–40^, the docking orientation was conserved compared to the wild-type complex, with a TCR-pMHC crossing angle of 64°, compared to 62° for the wild-type (Fig. 5a). Notably, there were minimal perturbations observed in the interface CDR loops or peptide (0.26 Å backbone atom RMSD for TCR CDR loops), indicating that our relatively conservative design strategy, involving specific point substitutions against a fixed pMHC structure, did not significantly alter the interface or proximal side chains (Fig. 6b–e).

**Fig. 7:**
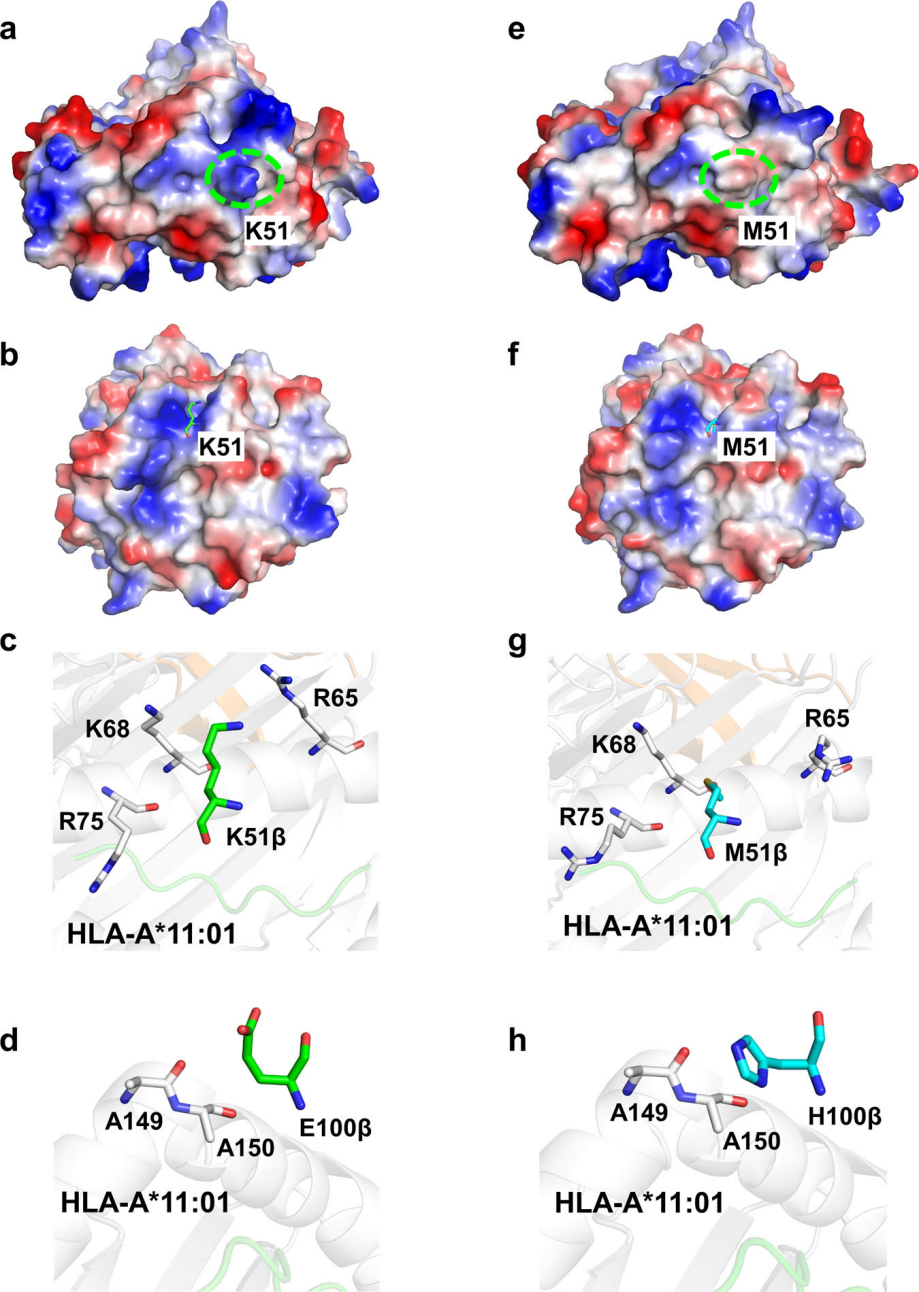
Surface Electrostatics of 4TCR2 WT/MH and KRAS^G12V^–HLA-A*11:01. **a** Surface electrostatics of 4TCR2-WT with the β chain K51 residue indicated by dotted green circle. **b** Surface electrostatics of pMHC in the 4TCR2-WT-KRAS^G12V^–HLA-A*11:01 complex. **c** Residues adjacent to TCR-β K51 on pMHC. **d** Residues adjacent to TCR-β E100 on pMHC. **e** Surface electrostatics of 4TCR2-MH with the β chain M51 residue indicated by dotted green circle. **f** Surface electrostatics of pMHC in the 4TCR2-MH-KRAS^G12V^–HLA-A*11:01 complex. **g** Residues adjacent to TCR-β M51 on pMHC. **h** Residues adjacent to TCR-β H100 on pMHC. Surface electrostatics: blue–positive, red-negative.

As anticipated in our modeling, the side chains of both methionine and histidine mutants within the βK51M/βE100H structure do not directly engage with the KRAS^G12V^ peptide and HLA-A*11:01. This finding implies that these residues might exert their influence indirectly by optimizing the residues that do establish direct interactions with the pMHC complex. Previous studies have illustrated that factors such as solvation states and improved electrostatic interactions can significantly impact TCR specificity and affinity towards antigenic peptides, even with minimal alterations in direct contacts^41^. Importantly, we observed a substantial disparity in the surface electrostatic potential surrounding the K51 mutation site between 4TCR2 MH and 4TCR2 WT (Fig. 7a–c, e–g). The βK51M mutation is situated at the periphery of the interface, adjacent to the positive electrostatic region formed by MHC residues R65, K68, and R75 (Fig. 7g). The substitution of a neutral residue (methionine; M) for a positively charged one (Lysine; K) in this position of the TCR is electrostatically permissible. As a result, we observe a shift towards a more negative electrostatic potential. This shift enhanced the contribution of these residues to the HLA-A*11:01 binding due to their predominantly positive electrostatic potential, resulting in a more complementary surface (Fig. 7). Furthermore, the mutant βE100H establishes hydrophobic contacts with the MHC residues A149 and A150 (Fig. 7d, h). Consequently, these mutations led to a 2% increase in the buried solvent accessible surface area for the pMHC, elevating it from 1676 Å^2^ to 1718 Å^2^.

To further understand the key factors responsible for the observed disparity in affinity, we conducted an analysis of the binding energy within the context of two complex structures: 4TCR2-WT-HLA-A*11:01-KRAS^G12V^ and 4TCR2-MH-HLA-A*11:01-KRAS^G12V^. This analysis revealed a notable divergence in binding energies amounting to 9.6 kcal/mol (Supplementary Table 5). Remarkably, the most significant contributor to this energy variation was identified as the solvation energy (fa_sol), exhibiting a marked disparity of 12.7 kcal/mol. Moreover, the repulsive van der Waals energy (fa_rep) and non-bonded electrostatic interactions also played substantial roles, contributing 6.5 kcal/mol and 1.0 kcal/mol, respectively, to the overall binding energy. These findings suggest that these factors might act in a compensatory manner, in response to changes in electrostatic properties in close proximity to the HLA residue near M51, which was corroborated by our examination of the complex structures.

## Discussion

The field of cancer immunotherapy has witnessed significant progress with the emergence of T cell-based therapies targeting tumor-specific neoantigens. Previous studies^42–45^ have highlighted the promise of this approach. In this study, our primary objective was to predict and screen neoantigens specific to mutant KRAS tumors that are presented by HLA-A*11:01. To achieve this, we conducted immunization experiments in HLA-A*11:01 transgenic mice, leading to the generation of mouse T cells and the isolation of TCRs that exhibited strong reactivity to the KRAS^G12V^ neoantigen. Subsequently, we employed a structure-based TCR design strategy to significantly enhance TCR affinity, achieving a remarkable 15-fold improvement with only two-point substitutions. The TCR variants identified in this study, with their enhanced affinity, hold significant potential for clinical applications in cancer immunotherapy.

The feasibility of identifying antigen-activated T cells through the prediction of tumor neoantigens is a promising strategy. KRAS, one of the most frequently mutated RAS isoforms in humans, is implicated in various cancers, including ~90% of pancreatic ductal adenocarcinomas (PDAC), 43% of colorectal cancers (CRC), 30–35% of non–small cell lung cancers (NSCLC), and 25–30% of lung adenocarcinomas^9,46–48^. Specifically, Gly12 point mutations in KRAS are highly prevalent, accounting for ~80% of KRAS-mutated malignancies, with 41% being G12D, 28% G12V, and 14% G12C^49,50^. Notably, KRAS^G12D^ and KRAS^G12V^ are the predominant mutations in PDAC and CRC, while KRAS^G12C^ is common in NSCLC. Additionally, other KRAS alleles, such as G12S and G13D, are mainly found in colorectal cancer^10,51^. Hence, our focus is on these KRAS mutations as potential tumor neoantigens for inducing CTL responses against these malignancies. To investigate this, we synthesized a set of twenty nonamer peptides and twelve decamer peptides, corresponding to both the wild type and five mutated forms of KRAS, which were predicted to bind to HLA-A*11:01 using the epitope prediction NetMHCpan4.0 algorithms (Supplementary Table 1). We then conducted experimental evaluations of the binding affinity between these candidate peptides and HLA-A*11:01 using UV-mediated peptide exchange assays. Our findings revealed that 10 peptides (31%) displayed strong binding capacity, while 2 peptides (6%) exhibited moderate binding. Notably, the majority of KRAS-derived peptides with robust HLA-A*11:01 binding capacity were found within the 7−16 and 8–16 regions, consistent with previous observations^27,52^. This assessment of HLA-A*11:01 binding capacity allowed us to narrow down the pool of predicted neoantigens, significantly reducing the workload for subsequent experiments. To access the immune response, we treated transgenic HLA-A*11:01 mice with mutated KRAS-derived peptides, namely KRAS_8–16_^G12V^, KRAS_8–16_^G12S^, or KRAS_7-16_^G13D^. Through single-cell sequencing, we identified a series of TCRs specific to KRAS_8–16_^G12V^. Furthmore, by in vitro refolding of the extracellular domain of the TCR, we successfully obtained the solubilized 4TCR2 protein.

The crystallographic analysis of the 4TCR2-HLA-A*11:01-KRAS^G12V^ complex has provided valuable mechanistic insights into neoantigen specificity. In comparison to the conformation of the KRAS^WT^ peptide in the pMHC (PDB: 8I5E), the KRAS^G12V^ peptide in the 4TCR2-bound form exhibited a significant conformational shift. Notable, peptide residue 6 in KRAS^G12V^ transitioned from an upward-facing exposed position to a downward-facing orientation. This conformational change also led to the formation of a network of interactions between the KRAS^G12V^ peptide and the HLA D-pocket. Furthermore, 4TCR2 docks onto HLA-A*11:01-KRAS^G12V^ in a canonical diagonal orientation. Specifically, the Vα domain is positioned over the α2 helix of HLA-A*11:01, while the Vβ domain is positioned over the α1 helix. However, it is worth noting that the crossing angles observed in comparison to previously reported TCR-pMHC complexes differ: 48° for 1–2C TCR(PDB: 8I5C), and 44° for 3-2E TCR (PDB: 8I5D)^26^ (Supplementary Fig. 6a, b). Additionally, these complexes exhibit variations in the incident angle, which corresponds to the degree of tilt of TCR over pMHC^53^. Specifically, the indicent angle is 8° for 4TCR2, 7° for 1–2 C TCR, and 12° for 3-2E TCR (Supplementary Fig. 6a).

Differing docking angles also manifest in the TCR interaction with peptides. In most previously examined TCR-pMHC complexes, both CDR3 loops engaged with the bound peptide. However, in the 4TCR2-HLA-A*11:01-KRAS^G12V^ complex all peptide interactions were exclusively mediated by the CDR3β loop. Specifically, residues R95 and S98 form hydrogen bonds with the P4 Ala and P6 Gly main chains of the peptide, respectively. Interestingly, R95 and S98 are positioned on opposite sides of the G12V mutation (P5 Val). Additionally, the side chain of another residue, D94, contributed to peptide recognition by contacting P8 Gly. In the 1-2C TCR structure, only Y96 in the TCR CDR3α region forms a hydrogen bond with V2 of the peptide (Supplementary Fig. 6c, e). In the structure of 3-2E TCR, which shares the same docking angle as 1–2C TCR, R50, located in the TCR CDR2β, engaged in a pair of hydrogen bonding interactions with G6 on the peptide (Supplementary Fig. 6d, f).

The affinity between TCR and pMHC is generally thought to play a crucial role in antigen recognition^54^. Since 4TCR2 exhibits a relatively weak affinity for pMHC, and because in vivo potency is somewhat linked to pMHC affinity, extensive efforts have been devoted to enhancing TCR affinity^35,40,52,55^. A major concern when striving to increase TCR affinity is maintaining peptide specificity. TCRs establish contacts with both peptide and MHC while recognizing peptides presented by MHC molecules. When making modifications to enhance TCR affinity, there is a risk of unintended cross-reactivity, especially if these modifications primarily target the MHC protein. Predicting such off-target interactions is challenging, as they cannot be reliably predicted solely from the peptide sequence. Notably, unanticipated cross-reactivity of a high-affinity TCR led to serious consequences, including fatalities, in a clinical trial^56^. Therefore, it is imperative to exercise meticulous control over both affinity and specificity in the development of enhanced TCRs for therapeutic applications.

Structure-based TCR design provides a strategy for enhancing TCR affinity towards pMHC while preserving its specificity for the desired antigen. Using this methodology, we were able to achieve a remarkable 15-fold affinity improvement in affinity through just two-point substitutions. Specifically, the 4TCR2-MH exhibited a *K*_*D*_ of 2 µM when bound to HLA-A*11:01-KRAS^G12V^ with no detectable binding affinity observed for HLA-A*11:01-KRAS^WT^ (Fig. 4b). This outcome underscores the enduring specificity of the affinity-enhanced 4TCR2-MH towards the KRAS^G12V^ neoantigen. Structure-based TCR design has previously been employed with success, as seen in the case of the A6 and DMF5 TCRs, resulting in variants with affinity increases of up to 100- and 400-fold increased affinity for pMHC, respectively^35,40^.

Remarkably, only two amino-acid substitutions, αD26Y and βL98W, yielded the most significant enhancement in affinity for the DMF5 TCR. Both the Tyr and Trp mutant side chains establish direct interactions with the peptide, forming more extensive contacts with the peptide than the wild-type TCR. However, in the structure of 4TCR2-MH-HLA-A*11:01-KRAS^G12V^, the side chains of the βK51M and βE100H mutations did not directly engage with the KRAS^G12V^ peptide. The augmented TCR affinity in 4TCR2-MH primarily stemmed from alterations in solvation states and improved electrostatic complementarity, with minimal changes in direct TCR-pMHC interactions. Although the affinity of 4TCR2-MH did not reach the magnitude of DMF5-YW, it did surpass the affinity threshold required for maximal T-cell activity. As a result, engineered T cells targeting the KRAS^G12V^ neoantigen with 4TCR2-MH hold significant promise as a potential cancer immunotherapy approach.

## Methods

### Peptide UV exchange and HLA-I ELISA

Peptides were reconstituted in deionized H_2_O to a concentration of 10 mM and stored at −80 °C. 25 μM positive control peptide (IVTDFSVIK), negative control peptide (NPKASLLSL) or KRAS-related peptides were prepared in 1× PBS. One volume of 1× PBS with or without peptides was mixed with one volume of 0.0125 mg/mL HLA-A*11:01 monomer on ice. Subsequently, the mixtures were exposed to 366 nm UV light for a duration of 30 minutes. The exchange efficiency was evaluated using an HLA class I ELISA kit. Briefly, UV-mediated HLA-peptide exchange products were diluted with 1× dilution buffer (100 mM NaCl, 50 mM Tris (pH 8.0), 0.1% BSA, 0.02% Tween 20). Following this, 100 µL of the diluted exchange product, the positive control, and the negative control were added to ELISA plates (BioLegend, Cat. 423501) pre-coated with streptavidin (BioLegend, Cat. 280302). The plates were sealed and incubated at 37 °C for 1 hour. Post-incubation, the plates were washed with 1× wash buffer (Biolegend, Cat. 421601). Subsequently, 100 microliters of diluted HRP-conjugated anti-human β2-microglobulin antibody (Biolegend, Cat. 280303) were added to the plates. The plates were sealed again and incubated at 37 °C for 1 hour, followed by another round of washing using 1× wash buffer. Then, the plates were exposed to a substrate solution (5.9 mM citric acid monohydrate, 4.1 mM trisodium citrate dihydrate, 0.08 mM ABTS, and 0.006% H2O2) for 8 minutes at room temperature. 50 µL of 2% (w/v) oxalic acid dihydrate in deionized water was added to each well to stop the chromogenic reaction. The optical density (OD) values at 405 nm were measured within 30 minutes. The percentage of positive signal was calculated by the following formula: percentage of positive signal = [(OD value of monomers with the predicted peptide−OD value of monomers with the negative control peptide)/(OD value of monomers with the positive control peptide−OD value of monomers with the negative control peptide)] ×100%.

### Mice

C57BL/6 mice were purchased from SPF (Beijing) Biotechnology Co., Ltd. Transgenic mice expressing the human HLA-A11:01 gene were obtained from Taconic Biosciences. Mice were housed and bred at the Institute of Microbiology, Chinese Academy of Sciences, in specific pathogen-free conditions. Both male and female mice were used for analysis and quantification. Sex-matched mice were used at 6–12 weeks old unless otherwise noted.

### Ethics statement

We have complied with all relevant ethical regulations for animal use. All animal experiments were approved by the Committee on the Ethics of Animal Experiments of the Institute of Microbiology, Chinese Academy of Science (IMCAS) and conducted in compliance with the recommendations in the Guide for the Care and Use of Laboratory Animals of IMCAS Ethics Committee.

### Antibody

In this study, the following fluorescent dye-conjugated antibodies were used: FITC-anti-mCD8a (53-6.7, cat. no. 100706, Biolegend), Percp-anti-mCD4 (RM4-5, cat. no. 100538, Biolegend); APC-anti-mIFNγ (XMG1.2, cat. no. 505810, Biolegend).

### Single-cell V(D)J + 5’ from CD8^+^ T cell using 10× genomics chromium and quality control of 10× Genomics single-cell RNA-seq

Sorted CD8^+^ T cells were loaded onto a 10× Genomics Chromium Chip per factory recommendations. Reverse transcription and library preparation were performed using the 10× Genomics Single Cell V(D)J + 5’ v3 kit following the 10× Genomics protocol. Libraries were sequenced on Illumina Novaseq6000 platform. Cells were removed if their gene expression <200 genes and >3500 genes or greater than 5% mitochondrial reads.

### Peptides

All KRAS-related peptides were purchased from Scilight-Peptide with purity >95%.

### Protein preparation

Soluble 4TCR2, which was used for affinity measurement and structure determination, was produced by in vitro folding from inclusion bodies expressed in *E. coli*, following the previously described method^57^. Codon-optimized genes encoding the TCR α chain (residues 1–198) and β chain (residues 1–240) were synthesized and cloned into the expression vector pET30a (GenScript). To enhance the folding yield of the TCR αβ heterodimer, an interchain disulfide bond (CαCys159–CβCys170) was introduced. The mutated α and β chains were expressed separately as inclusion bodies in BL21(DE3) *E. coli* cells. Bacteria were grown at 37 °C in LB medium until reaching an OD_600_ of 0.6–0.8, then induced with 1 mM isopropyl-β-D-thiogalactoside. After 4 hours of incubation, the bacteria were harvested by centrifugation and resuspended in a solution containing 50 mM Tris-HCl (pH 8.0), 25% sucrose, 10 mM DTT, and 1 mM EDTA. Cell disruption was achieved through sonication, and the inclusion bodies were washed with a solution containing 50 mM Tris-HCl (pH 8.0), 1% (v/v) Triton X-100, 200 mM NaCl, and 1 mM EDTA. Subsequently, the inclusion bodies were dissolved in a denaturing solution consisting of 6 M guanidinium chloride, 50 mM Tris-HCl (pH 8.1), 10 mM EDTA, and 10 mM DTT. For in vitro folding, the TCR α chain (38 mg) and β chain (47 mg) were mixed and diluted into a 1-liter folding buffer containing 5 M urea, 0.4 M L-arginine-HCl, 100 mM Tris-HCl (pH 8.1), 5 mM reduced glutathione, and 0.5 mM oxidized glutathione. The refolding mixture was dialyzed twice against 10 volumes of 20 mM Tris (pH 8.1). Correctly folded protein was purified using anion exchange chromatography and size exclusion chromatography, following previously established protocols.

Soluble HLA-A*11:01 loaded with either the wild-type KRAS peptide (VVGAGGVGK) or the mutant KRAS peptide (VVGAVGVGK) was prepared by in vitro folding of *E. coli* inclusion bodies, as described by Rodenko et al.^58^. The inclusion bodies were dissolved in a solution containing 8 M urea, 50 mM MES (pH 6.5), 1 mM EDTA, and 1 mM DTT. For in vitro folding, the HLA-A*11:01 heavy chain (34 mg), β2-microglobulin (25 mg), and either the wild-type or mutant KRAS peptide (10 mg) were mixed and added dropwise to 1 liter of ice-cold folding buffer containing 0.4 M L-arginine, 100 mM Tris-HCl (pH 8.0), 2 mM EDTA, 5 mM reduced glutathione, and 0.5 mM oxidized glutathione. The folding mixture was concentrated and purified using consecutive Superdex 200 chromatography steps with a buffer containing 20 mM Tris-HCl (pH 8.0) and 150 mM sodium chloride.

### Crystallization and protein structure determination

For the crystallization of the 4TCR2–KRAS_8–16_^G12V^–HLA-A*11:01 complex, we mixed 4TCR2 with KRAS_8–16_^G12V^–HLA-A*11:01 in a 1:1 molar ratio at a concentration of 10 mg/mL. Crystals were obtained at 16 °C using vapor diffusion in sitting drops. The 4TCR2–KRAS_8–16_^G12V^–HLA-A*11:01 complex crystallized in a solution containing 20% (w/v) PEG 3350 and 0.2 M sodium citrate. The 4TCR2-MH–KRAS_8–16_^G12V^–HLA-A*11:01 complex was grown under the following conditions: 20% (w/v) PEG 3350 and 0.2 M ammonium citrate tribasic at pH 7.0. To collect the data, the crystals were cryoprotected with a 10% (w/v) glycerol solution and flash-cooled. Diffraction data for the 4TCR2–KRAS_8–16_^G12V^–HLA-A*11:01 complex were collected at a wavelength of 0.97918 Å on BL02U1 at the Shanghai Synchrotron Radiation Facility (SSRF), while the 4TCR2-MH–KRAS_8–16_^G12V^–HLA-A*11:01 data were collected at a wavelength of 1.03845 Å on BL18U1. The data were processed and scaled using the HKL3000 package and autoPROC^59^. The structures were determined using the molecular replacement (MR) method in the PHASER program^60^, with the structure of HLA-A*11:01 and β2m (PDB: 5WJL)^61^, and a murine P14 TCR (PDB: 6G9Q) used as the initial search model. The model was built into the modified experimental electron density using COOT^62^ and further refined in PHENIX^63^. The final refinement statistics are summarized in Supplementary Table 4. Structural figures were prepared using PyMOL.

### Simulation and scoring of 4TCR2 point mutations

We used PyRosetta to model point mutations of the TCR, as previously described^40^. PyRosetta was initialized with a command-line flag to include additional amino acid rotamers in the design process (init(extra_options = ‘extrachi_cutoff 1 -ex1 -ex2 -ex3’)). During the design process, only the mutant side chain was repacked, while the protein backbone from the wild-type structure was preserved. Point mutations were modeled in selected regions of the TCR, where residues were within 8 Å of the peptide presented by the MHC molecule. We scanned all 20 natural amino acids, except for cysteine, at each selected position and calculated the binding score. For a simple design, we used the default score function REF2015 during the entire design process. We first scored the complex, then isolated and scored the TCR and pMHC separately. The binding score was calculated by subtracting the TCR and pMHC scores from the complex. To improve the designs, we further refined the backbone of the TCR complementarity determining region loops through a combination of cyclic coordinate descent (CCD) and Monte Carlo algorithms. Finally, the designed mutations were ranked by the resulting change in the binding score.

### Surface plasmon resonance analysis

The interaction between 4TCR2 and KRAS_8–16_^WT^–HLA-A*11:01, as well as KRAS_8–16_^G12V^–HLA-A*11:01, was evaluated using surface plasmon resonance (SPR) on a Biacore 8 K instrument (Cytiva Life Sciences, USA) at a temperature of 25 °C. The KRAS_8–16_^WT^–HLA-A*11:01 and KRAS_8–16_^G12V^–HLA-A*11:01 molecules were immobilized on CM5 biosensor chips (GE Healthcare) at a density of 2000 resonance units (RU). An additional flow cell was injected with free biotin alone to serve as a blank control. To analyze the binding of the TCR, solutions containing varying concentrations of 4TCR2 or 4TCR2 mutants were sequentially flowed over the chips with immobilized KRAS_8–16_^WT^–HLA-A*11:01 and KRAS_8–16_^G12V^–HLA-A*11:01. Both equilibrium and kinetic data were fitted using a 1:1 binding model with BIA Evaluation 3.1 software.

### Statistics and reproducibility

Prism 8 software is used for statistical analysis. *T* test was performed for two-group analysis. *P* value <0.05 was considered as statistically significant. The number of replicates was specified in the figure legends.

### Supplementary information

Supplementary Information

Description of additional supplementary files

Supplementary Data 1

## Data Availability

The Structure coordinates and reflections have been deposited in the protein data bank under accession numbers 8WTE and 8WUL. Source data for figures and tables can be found in Supplementary Data 1. Further information and requests for resources should be directed to and will be fulfilled by the lead contact, Sheng Ye (sye@tju.edu.cn).

## References

[1] Downward J (2003). Targeting ras signalling pathways in cancer therapy. Nat. Rev. Cancer.

[2] Drugan JK, Rogers-Graham K, Gilmer T, Campbell S, Clark GJ (2000). The Ras/p120 GTPase-activating protein (GAP) interaction is regulated by the p120 GAP pleckstrin homology domain. J. Biol. Chem..

[3] Bos JL, Rehmann H, Wittinghofer A (2007). GEFs and GAPs: critical elements in the control of small G proteins. Cell.

[4] Huang, L. M., Guo, Z. X., Wang, F. & Fu, L. W. KRAS mutation: from undruggable to druggable in cancer. *Sig. Transduct. Target Ther.* **6**, 10.1038/s41392-021-00780-4 (2021). 10.1038/s41392-021-00780-4 PMC8591115 34776511

[5] Prior IA, Hood FE, Hartley JL (2020). The frequency of ras mutations in cancer. Cancer Res..

[6] Cerami E (2012). The cBio cancer genomics portal: an open platform for exploring multidimensional cancer genomics data. Cancer Discov..

[7] Consortium APG (2017). AACR Project GENIE: powering precision medicine through an international consortium. Cancer Discov..

[8] Cox AD, Fesik SW, Kimmelman AC, Luo J, Der CJ (2014). Drugging the undruggable RAS: mission possible?. Nat. Rev. Drug Discov..

[9] Fernandez-Medarde A, Santos E (2011). Ras in cancer and developmental diseases. Genes Cancer.

[10] Hofmann MH, Gerlach D, Misale S, Petronczki M, Kraut N (2022). Expanding the reach of precision oncology by drugging all KRAS mutants. Cancer Discov..

[11] Chen H, Smaill JB, Liu TZ, Ding K, Lu XY (2020). Small-molecule inhibitors directly targeting KRAS as anticancer therapeutics. J. Med Chem..

[12] Canon J (2019). The clinical KRAS(G12C) inhibitor AMG 510 drives anti-tumour immunity. Nature.

[13] Fell JB (2020). Identification of the clinical development candidate MRTX849, a covalent KRAS(G12C) inhibitor for the treatment of cancer. J. Med Chem..

[14] Hong DS (2020). KRAS(G12C) inhibition with sotorasib in advanced solid tumors. N. Engl. J. Med.

[15] Christensen JG, Hallin J (2022). The KRAS(G12D) inhibitor MRTX1133 elucidates KRAS-mediated oncogenesis. Nat. Med..

[16] Hallin J (2022). Anti-tumor efficacy of a potent and selective non-covalent KRAS(G12D) inhibitor. Nat. Med..

[17] Issahaku, A. R. et al. Characterization of the binding of MRTX1133 as an avenue for the discovery of potential KRAS(G12D) inhibitors for cancer therapy. *Sci. Rep.* **12**, 10.1038/s41598-022-22668-1 (2022). 10.1038/s41598-022-22668-1 PMC9588042 36273239

[18] Mao ZW (2022). KRAS(G12D) can be targeted by potent inhibitors via formation of salt bridge. Cell Discov..

[19] Chatani PD, Yang JC (2020). Mutated RAS: targeting the “untargetable” with T cells. Clin. Cancer Res..

[20] Shafer P, Kelly LM, Hoyos V (2022). Cancer therapy with TCR-engineered T cells: current strategies, challenges, and prospects. Front. Immunol..

[21] Lowe KL (2019). Novel TCR-based biologics: mobilising T cells to warm ‘cold’ tumours. Cancer Treat. Rev..

[22] Middleton, D., Menchaca, L., Rood, H. & Komerofsky, R. New allele frequency database: http://www.allelefrequencies.net. *Tissue Antigens* **61**, 403–407 (2003). 10.1034/j.1399-0039.2003.00062.x 12753660

[23] Shen Y (2014). Distribution of HLA-A, -B, and -C alleles and HLA/KIR combinations in Han population in China. J. Immunol. Res..

[24] Jurtz V (2017). NetMHCpan-4.0: improved peptide-MHC class I interaction predictions integrating eluted ligand and peptide binding affinity data. J. Immunol..

[25] Huang M (2016). Improved transgenic mouse model for studying HLA class I antigen presentation. Sci. Rep..

[26] Lu D (2023). KRAS G12V neoantigen specific T cell receptor for adoptive T cell therapy against tumors. Nat. Commun..

[27] Wang QJ (2016). Identification of T-cell receptors targeting KRAS-mutated human tumors. Cancer Immunol. Res..

[28] Rudolph MG, Stanfield RL, Wilson IA (2006). How TCRs bind MHCs, peptides, and coreceptors. Annu Rev. Immunol..

[29] Kammertoens T, Blankenstein T (2013). It’s the peptide-MHC affinity, stupid. Cancer Cell.

[30] Zhong S (2013). T-cell receptor affinity and avidity defines antitumor response and autoimmunity in T-cell immunotherapy. Proc. Natl. Acad. Sci. USA.

[31] Spear TT, Evavold BD, Baker BM, Nishimura MI (2019). Understanding TCR affinity, antigen specificity, and cross-reactivity to improve TCR gene-modified T cells for cancer immunotherapy. Cancer Immunol. Immunother..

[32] Tan MP (2015). T cell receptor binding affinity governs the functional profile of cancer-specific CD8+ T cells. Clin. Exp. Immunol..

[33] Schmid DA (2010). Evidence for a TCR affinity threshold delimiting maximal CD8 T cell function. J. Immunol..

[34] Oren R (2014). Functional comparison of engineered T cells carrying a native TCR versus TCR-like antibody-based chimeric antigen receptors indicates affinity/avidity thresholds. J. Immunol..

[35] Haidar JN (2009). Structure-based design of a T-cell receptor leads to nearly 100-fold improvement in binding affinity for pepMHC. Proteins.

[36] Sami M (2007). Crystal structures of high affinity human T-cell receptors bound to peptide major histocompatibility complex reveal native diagonal binding geometry. Protein Eng. Des. Sel..

[37] Dunn SM (2006). Directed evolution of human T cell receptor CDR2 residues by phage display dramatically enhances affinity for cognate peptide-MHC without increasing apparent cross-reactivity. Protein Sci..

[38] Jones LL (2008). Different thermodynamic binding mechanisms and peptide fine specificities associated with a panel of structurally similar high-affinity T cell receptors. Biochemistry.

[39] Madura F (2013). T-cell receptor specificity maintained by altered thermodynamics. J. Biol. Chem..

[40] Pierce BG (2014). Computational design of the affinity and specificity of a therapeutic T cell receptor. PLoS Comput. Biol..

[41] Crean RM (2020). Molecular rules underpinning enhanced affinity binding of human T cell receptors engineered for immunotherapy. Mol. Ther. Oncolytics.

[42] Tran E (2016). T-cell transfer therapy targeting mutant KRAS in cancer. N. Engl. J. Med..

[43] Schumacher TN, Scheper W, Kvistborg P (2019). Cancer neoantigens. Annu. Rev. Immunol..

[44] Parkhurst MR (2019). Unique neoantigens arise from somatic mutations in patients with gastrointestinal cancers. Cancer Discov..

[45] Leidner R (2022). Neoantigen T-cell receptor gene therapy in pancreatic cancer. N. Engl. J. Med..

[46] Eser S, Schnieke A, Schneider G, Saur D (2014). Oncogenic KRAS signalling in pancreatic cancer. Br. J. Cancer.

[47] Neumann J, Zeindl-Eberhart E, Kirchner T, Jung A (2009). Frequency and type of KRAS mutations in routine diagnostic analysis of metastatic colorectal cancer. Pathol. Res. Pract..

[48] Biankin AV (2012). Pancreatic cancer genomes reveal aberrations in axon guidance pathway genes. Nature.

[49] Karachaliou N (2013). KRAS mutations in lung cancer. Clin. Lung Cancer.

[50] Prior IA, Lewis PD, Mattos C (2012). A comprehensive survey of Ras mutations in cancer. Cancer Res..

[51] Gao Q (2020). Selective targeting of the oncogenic KRAS G12S mutant allele by CRISPR/Cas9 induces efficient tumor regression. Theranostics.

[52] Poole A (2022). Therapeutic high affinity T cell receptor targeting a KRAS(G12D) cancer neoantigen. Nat. Commun..

[53] Pierce BG, Weng Z (2013). A flexible docking approach for prediction of T cell receptor-peptide-MHC complexes. Protein Sci..

[54] Tian S, Maile R, Collins EJ, Frelinger JA (2007). CD8+ T cell activation is governed by TCR-peptide/MHC affinity, not dissociation rate. J. Immunol..

[55] Li Y (2005). Directed evolution of human T-cell receptors with picomolar affinities by phage display. Nat. Biotechnol..

[56] Linette GP (2013). Cardiovascular toxicity and titin cross-reactivity of affinity-enhanced T cells in myeloma and melanoma. Blood.

[57] Boulter JM (2003). Stable, soluble T-cell receptor molecules for crystallization and therapeutics. Protein Eng..

[58] Rodenko B (2006). Generation of peptide-MHC class I complexes through UV-mediated ligand exchange. Nat. Protoc..

[59] Minor W, Cymborowski M, Otwinowski Z, Chruszcz M (2006). HKL-3000: the integration of data reduction and structure solution–from diffraction images to an initial model in minutes. Acta Crystallogr. D. Biol. Crystallogr..

[60] McCoy AJ (2007). Phaser crystallographic software. J. Appl. Crystallogr..

[61] Culshaw A (2017). Germline bias dictates cross-serotype reactivity in a common dengue-virus-specific CD8(+) T cell response. Nat. Immunol..

[62] Emsley P, Lohkamp B, Scott WG, Cowtan K (2010). Features and development of Coot. Acta Crystallogr. D. Biol. Crystallogr..

[63] Adams PD (2010). PHENIX: a comprehensive Python-based system for macromolecular structure solution. Acta Crystallogr. D. Biol. Crystallogr..

