# Supplementary material for: Identification and affinity enhancement of T-cell receptor targeting a KRASG12V cancer neoantigen
Source: Commun Biol. 2024 Apr 29;7:512. doi: 10.1038/s42003-024-06209-2 (PMC11058820; doi:10.1038/s42003-024-06209-2)
Supplement: Supplementary file 1 — Supplementary Information [file 42003_2024_6209_MOESM1_ESM.pdf]

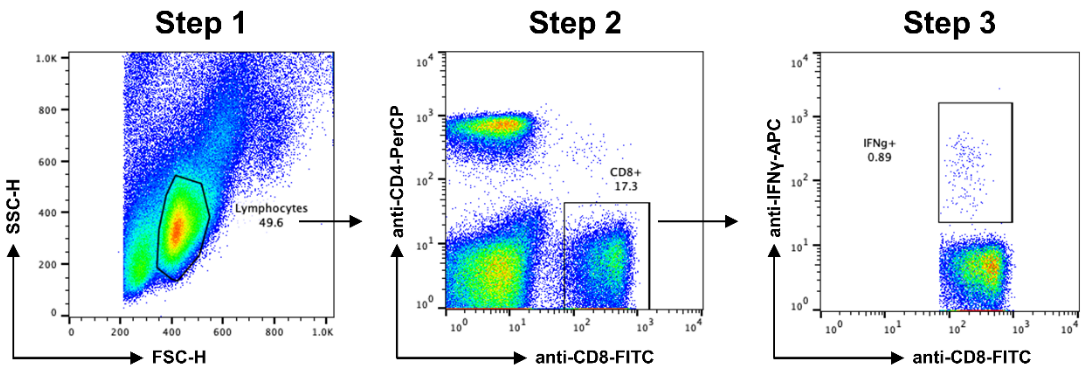

Supplementary Figure 1: Flow cytometry gating strategy for IFNγ staining of antigen-specific CD8⁺ T cells. Step 1: defining single cell populations with FSC and SSC; **Step 2**: gating CD8⁺ T cells with anti-CD8-FITC antibody and anti-CD4-PerCP antibody; **Step 3**: staining of IFNγ⁺ CD8⁺ T cells with anti-IFNγ-APC antibody.

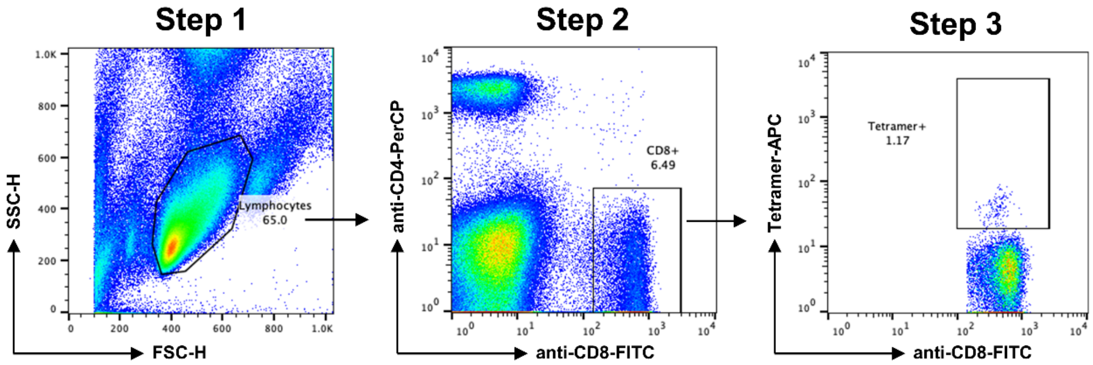

Supplementary Figure 2: Flow cytometry gating strategy for antigen-specific staining of KRAS-specific CD8$^+$ T cells. Step 1: defining single cell populations with FSC and SSC; **Step 2**: gating of CD8$^+$ T cells with anti-CD8-FITC antibody and anti-CD4-PerCP antibody; **Step 3**: staining of KRAS-specific CD8$^+$T cells by pMHC Tetramer-APC.

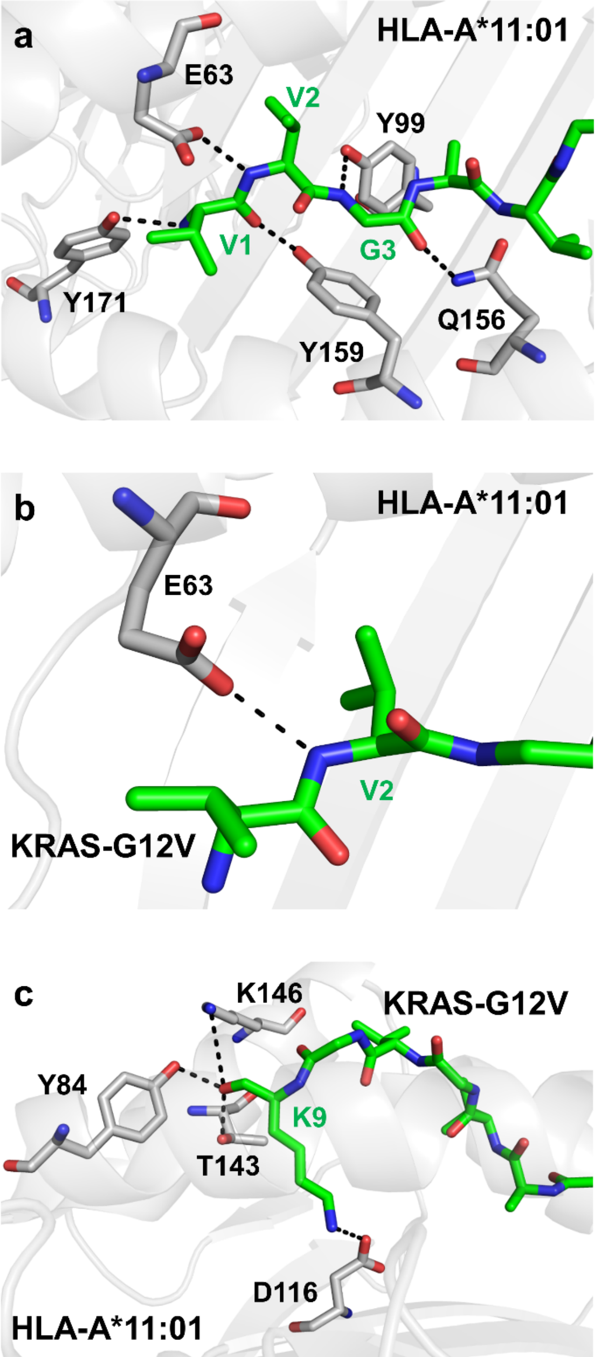

Supplementary Figure 3: The neoantigen KRAS^{G12V} binds to HLA-A*11:01 in a canonical fashion. a Bird's-eye view of the N-terminal KRAS^{G12V} peptide interactions with HLA-A*11:01. The peptide (green) and the side chains (gray) of interacting residues of HLA-A*11:01 are represented as sticks. Hydrogen bonds are shown as dashed lines. **b** The peptide residue V2 and HLA-A*11:01 E63 formed hydrogen bond. **C** The HLA interaction network around the peptide residue K9 in the 4TCR2-HLA-A*11:01-KRAS^{G12V} complex.

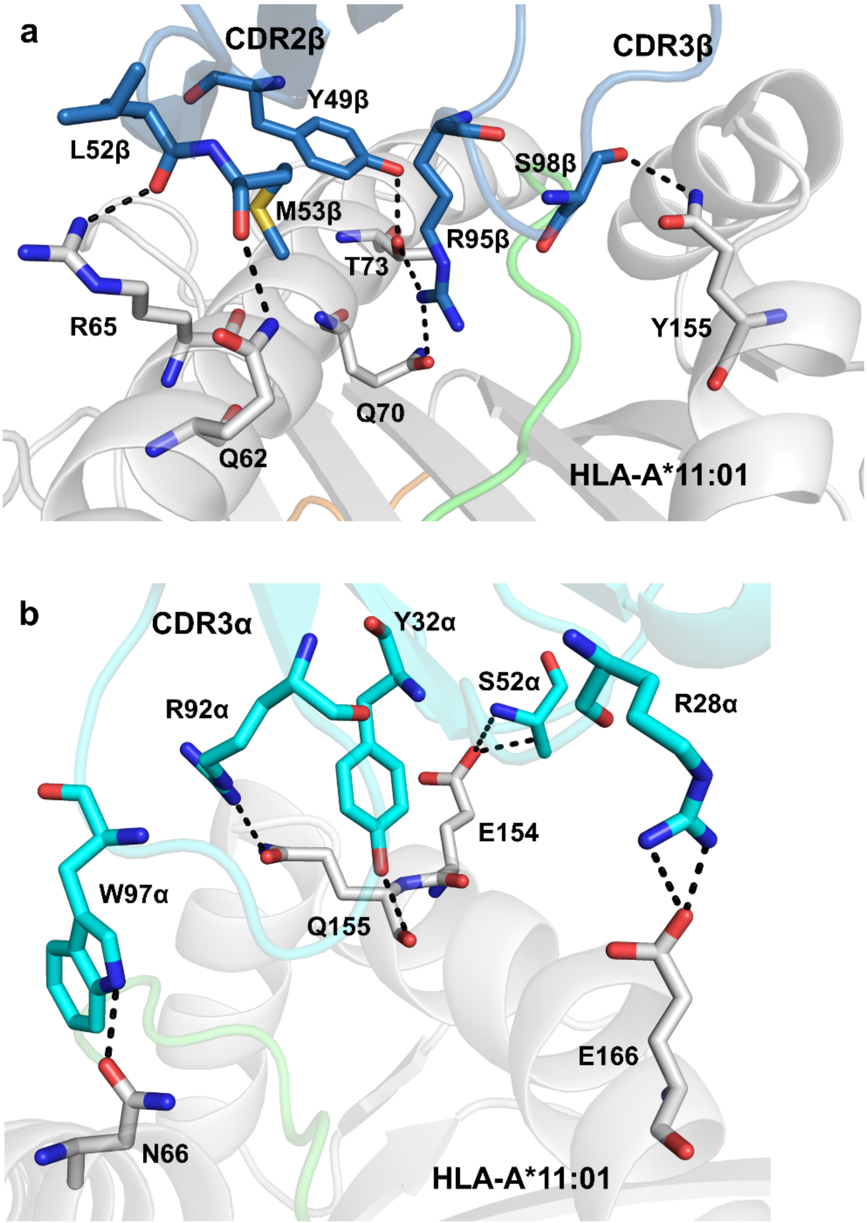

Supplementary Figure 4: TCR and HLA interaction network in the 4TCR2-HLA-A*11:01-KRAS^{G12V} complex. **a** Interactions between the 4TCR2β CDRs and HLA. **b** Interactions between the 4TCR2α CDRs and HLA. The TCR and HLA residues that are up to 4 Å of each other are shown in sticks. The dotted lines indicate polar contacts. The KRAS^{G12V} peptide residues are shown as green cartoon.

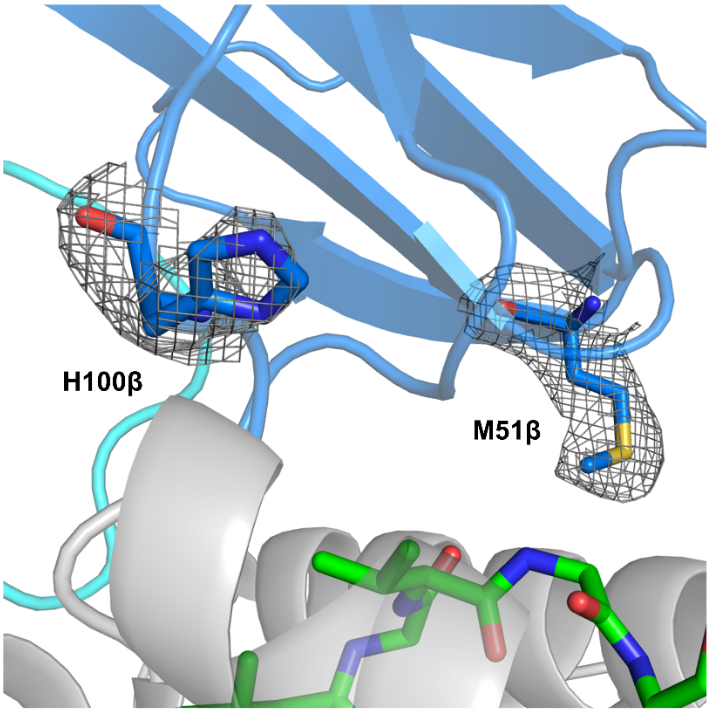

Supplementary Figure 5: Electron density for βM51 and βH100 in the 4TCR2 MH-HLA-A*11:01-KRAS^{G12V} crystal structure contoured at 1σ calculated from an unbiased, iterative-build OMIT map. The density shows the clear, unambiguous positioning of the two mutated residues.

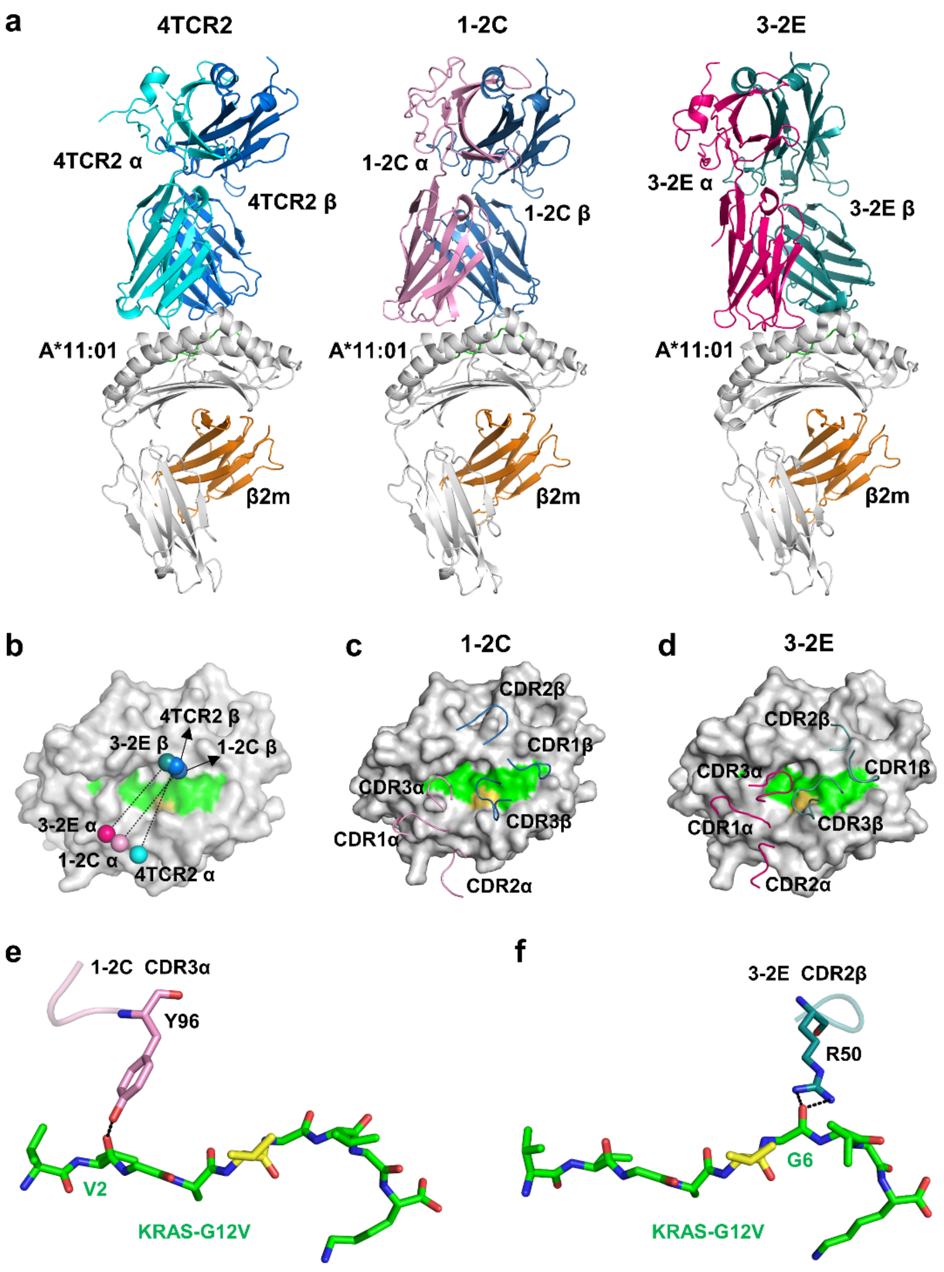

Supplementary Figure 6: Comparison of 4TCR2 with 1-2C and 3-2E. a Structure of the 4TCR2, 1-2C and 3-2E TCRs bound to HLA-A*11:01-KRAS^{G12V}. **b** The binding orientation of 4TCR2, 1-2C and 3-2E TCRs with HLA-A*11:01-KRAS^{G12V}. The black dashed line indicates the crossing angle of TCR to pMHC. HLA-A*11:01 is depicted as a light gray surface. The KRAS^{G12V} peptide is presented as a surface in green with the mutated P5 Val residue in yellow. **c** Positions of CDR loops of 1-2C TCR on HLA-A*11:01-KRAS^{G12V} (top view). CDRs of 1-2C are shown as numbered pink (CDR1α, CDR2α, and CDR3α) or skyblue (CDR1β, CDR2β, and CDR3β) loops. **d** Positions of CDR loops of 3-2E TCR on HLA-A*11:01-KRAS^{G12V} (top view). CDRs of 3-2E are shown as numbered hot pink (CDR1α,

CDR2α, and CDR3α) or deepteal (CDR1β, CDR2β, and CDR3β) loops. **e** Interactions of the 1-2C TCR with the $KRAS^{G12V}$ peptide. Hydrogen bond is represented by black dashed lines. **f** Interactions of the 3-2E TCR with the KRAS-G12V peptide.

Supplementary Table 1: KRAS-derived peptides table. KRAS-derived, predicted neoantigens identified by the Promiscuous MHC Binding Peptide Prediction Server, respectively.

Name	Length	Sequence
$KRAS_{5-13}$	9	KLVVVGAGG
$KRAS_{5-13}$ G12D	9	KLVVVGADG
$KRAS_{5-13}$ G12S	9	KLVVVGASG
$KRAS_{5-13}$ G12V	9	KLVVVGAVG
$KRAS_{5-14}$	10	KLVVVGAGGV
$KRAS_{5-14}$ G12C	10	KLVVVGACGV
$KRAS_{5-14}$ G12D	10	KLVVVGADGV
$KRAS_{5-14}$ G12S	10	KLVVVGASGV
$KRAS_{5-14}$ G12V	10	KLVVVGAVGV
$KRAS_{5-14}$ G13D	10	KLVVVGAGDV
$KRAS_{6-14}$	9	LVVVGAGGV
$KRAS_{6-14}$ G12C	9	LVVVGACGV
$KRAS_{6-14}$ G12D	9	LVVVGADGV
$KRAS_{6-14}$ G12S	9	LVVVGASGV
$KRAS_{6-14}$ G12V	9	LVVVGAVGV
$KRAS_{6-14}$ G13D	9	LVVVGAGDV
$KRAS_{7-15}$	9	VVVGAGGVG
$KRAS_{7-15}$ G12D	9	VVVGADGVG
$KRAS_{7-15}$ G12S	9	VVVGASGVG
$KRAS_{7-15}$ G12V	9	VVVGAVGVG
$KRAS_{7-16}$	10	VVVGAGGVGK
$KRAS_{7-16}$ G12C	10	VVVGACGVGK
$KRAS_{7-16}$ G12D	10	VVVGADGVGK
$KRAS_{7-16}$ G12S	10	VVVGASGVGK
$KRAS_{7-16}$ G12V	10	VVVGAVGVGK
$KRAS_{7-16}$ G13D	10	VVVGAGDVGK
$KRAS_{8-16}$	9	VVGAGGVGK
$KRAS_{8-16}$ G12C	9	VVGACGVGK
$KRAS_{8-16}$ G12D	9	VVGADGVGK
$KRAS_{8-16}$ G12S	9	VVGASGVGK
$KRAS_{8-16}$ G12V	9	VVGAVGVGK
$KRAS_{8-16}$ G13D	9	VVGAGDVGK

Supplementary Table 2: $KRAS^{G12V}$ specific $CD8^+$ T cells clonotypes information

Clonotype	TRA-CDRs	TRB-CDRs
TCR1	CAVTGSGGKLTL	CASSRWGGDTQYF
TCR2	CAARSSGSWQLIF	CASSQDRGDSAETLYF
TCR3	CAVSKVVF<br>CAASEQGNYAQGLTF	CASSPQNSDYTF
TCR4	CAVGSAGNKLTF	CASSNWGDEQYF
TCR5	CAARDSNYQLIW	CASGDTGGYEQYF

Supplementary Table 3: KRAS^{G12V}-reactive TCR germline genes and CDR3 sequences

Name	TRAV	TRAJ	CDR3α	TRBV	TRBJ	CDR3β
4TCR2	7D-2*01	22	CAARSSGSWQLIF	2*01	2-3	CASSQDRGDSAETLYF

Supplementary Table 4: Data collection and refinement statistics

	**4TCR2-WT-HLA-A*11:01-KRAS^{G12V}**	**4TCR2-MH-HLA-A*11:01-KRAS^{G12V}**
**PDB code**	8WTE	8WUL
**Data collection**		
Space group	P1	P1
Cell dimensions (Å)		
a, *b*, *c* (Å)	45.6, 96.1, 117.8	87.8, 96.3, 123.1
α, β, γ (°)	80.8, 82.1, 78.6	94.6, 90.6, 93.2
Resolution (Å)	115.59-2.17(2.29-2.17)[a]	30-2.36 (2.40-2.36)
R_{merge} (%)	7.2 (35.9)	5.9 (43.0)
$I / \sigma I$	5.3 (2.2)	27.1 (1.7)
Completeness (%)	96.0 (86.5)	98.1 (96.6)
Redundancy	2.7 (2.5)	3.4 (3.1)
**Refinement**		
Resolution (Å)	39.01-2.17	29.7-2.4
No. reflections	97700	161,533
R_{work} / R_{free} (%)	17.1/21.6	19.0/23.5
No. atoms		
Protein	12926	25613
Ligand/ion	0	0
Water	505	403
B-factors		
Protein	53.1	74.63
Ligand/ion	0	0
Water	48.6	62.02
R.m.s. deviations		
Bond lengths (Å)	0.005	0.002
Bond angles (°)	0.72	0.50

[a] Values in parentheses are for highest-resolution shell.

Supplementary Table 5: Differences in contribution to the binding energy between 4TCR2-WT and 4TCR2-MH bound TCR-pMHC complexes

Term[a]	Weight	4TCR2-WT +pMHC	4TCR2-MH +pMHC	Difference[b]	Units
fa_atr	1	-65.555	-54.84	10.715	kcal/mol
fa_rep	0.55	11.885	5.309	-6.576	kcal/mol
fa_sol	1	49.166	36.419	-12.747	kcal/mol
fa_intra_rep	0.005	0	0.001	0.001	kcal/mol
fa_intra_sol_xover4	1	0	0.001	0.001	kcal/mol
lk_ball_wtd	1	0.278	0.056	-0.222	kcal/mol
fa_elec	1	-17.343	-18.312	-0.969	kcal/mol
pro_close	1.25	0	0	0	AU[c]
hbond_sr_bb	1	0	0	0	kcal/mol
hbond_lr_bb	1	0	0	0	kcal/mol
hbond_bb_sc	1	-4.608	-3.599	1.009	kcal/mol
hbond_sc	1	-3.043	-3.894	-0.851	kcal/mol
dslf_fa13	1.25	-0.001	0	0.001	kcal/mol
omega	0.4	-0.001	0.001	0.002	AU
fa_dun	0.7	0	-0.001	-0.001	*kT*
p_aa_pp	0.6	0	0	0	*kT*
yhh_planarity	0.625	0	0	0	AU
ref	1	0	0	0	AU
rama_prepro	0.45	0	0	0	*kT*
total energy		-29.222	-38.859		

[a] Items are names of energy terms in the Rosettacode.
[b] Differences were calculated by subtracting the 4TCR2-WT-HLA-A*11:01-KRAS^{G12V} values from the 4TCR2-MH -HLA-A*11:01-KRAS^{G12V} values.
[c] AU = arbitrary units.
