# Supplementary material for: Identification and affinity enhancement of T-cell receptor targeting a KRASG12V cancer neoantigen
Source: Commun Biol. 2024 Apr 29;7:512. doi: 10.1038/s42003-024-06209-2 (PMC11058820; doi:10.1038/s42003-024-06209-2)
Supplement: Supplementary file 2 — Description of additional supplementary files [file 42003_2024_6209_MOESM2_ESM.pdf]

Description of Additional Supplementary Files

File name: Supplementary Data 1

Description: The source data behind the Figure 1b, Figure 4 and Table 1 in the paper.
